# A Single Subcutaneous Injection of Cellulose Ethers Administered Long before Infection Confers Sustained Protection against Prion Diseases in Rodents

**DOI:** 10.1371/journal.ppat.1006045

**Published:** 2016-12-14

**Authors:** Kenta Teruya, Ayumi Oguma, Keiko Nishizawa, Maki Kawata, Yuji Sakasegawa, Hiroshi Kamitakahara, Katsumi Doh-ura

**Affiliations:** 1 Department of Neurochemistry, Tohoku University Graduate School of Medicine, Sendai, Miyagi, Japan; 2 Division of Forest and Biomaterials Science, Graduate School of Agriculture, Kyoto University, Sakyo-ku, Kyoto, Japan; Istituto Superiore di Sanità, ITALY

## Abstract

Prion diseases are fatal, progressive, neurodegenerative diseases caused by prion accumulation in the brain and lymphoreticular system. Here we report that a single subcutaneous injection of cellulose ethers (CEs), which are commonly used as inactive ingredients in foods and pharmaceuticals, markedly prolonged the lives of mice and hamsters intracerebrally or intraperitoneally infected with the 263K hamster prion. CEs provided sustained protection even when a single injection was given as long as one year before infection. These effects were linked with persistent residues of CEs in various tissues. More effective CEs had less macrophage uptake ratios and hydrophobic modification of CEs abolished the effectiveness. CEs were significantly effective in other prion disease animal models; however, the effects were less remarkable than those observed in the 263K prion-infected animals. The genetic background of the animal model was suggested to influence the effects of CEs. CEs did not modify prion protein expression but inhibited abnormal prion protein formation in vitro and in prion-infected cells. Although the mechanism of CEs in vivo remains to be solved, these findings suggest that they aid in elucidating disease susceptibility and preventing prion diseases.

## Author Summary

Prion diseases are progressive, fatal, neurodegenerative transmissible illnesses in humans and animals caused by prion accumulation in the brain and lymphoreticular system. Because they are prevalent in nature, with atypical forms continuing to emerge, prion diseases are potential threats to both public health and the economy. However, there are no effective methods to prevent these diseases. Here we report that cellulose ethers (CEs), which are non-digestible water-soluble polysaccharides that are commonly used as inactive ingredients in foods and pharmaceuticals, show prophylactic efficacy in prion-infected animals. CEs persist in various tissues and confer sustained preventive efficacy for years, suggesting that they help to prevent prion diseases. Although the enteral absorption of CEs is limited, we found that a portion of the absorbed CEs influences disease progression. Therefore, CEs may be useful to assess disease susceptibility and prevent disease occurrence.

## Introduction

Prion diseases or transmissible spongiform encephalopathies are fatal neurodegenerative conditions caused by prion accumulation in the brain and lymphoreticular system [1]. Creutzfeldt–Jakob disease is the most common human prion disease that sporadically occurs mostly in the elderly. The number of cases of human prion disease is very small at a few cases per million persons, but the prevalence has been gradually increasing in accordance with the aging of society [2]. Despite recent tremendous therapeutic developments [3–6], remedies or preventive measures to inhibit disease progression or achieve significantly beneficial improvements have not yet been established.

In animal prion diseases such as scrapie in sheep and bovine spongiform encephalopathy in cattle, not only classical but also atypical, cases are known to sporadically occur [7]. In addition, cervine prion diseases such as chronic wasting disease are prevalent in domesticated as well as wild animals [8]. These animal prion diseases have become potential threats to public health and the economy. In particular, the issue of chronic wasting disease in wild animals is serious because affected animals or prion-carriers are difficult to eliminate, prions of chronic wasting disease are shed into excreta, and prions resist decomposition in soil and cadavers [8]. No means for preventing these animal diseases have been established, and development of prophylactic measures such as vaccines has long been awaited. However, prions are not efficiently eliminated by the immune system, which has stalled efforts to develop safe effective vaccines [9–11].

In our early study on the development of a certain anti-prion compound, we noticed that not only anti-prion compound-containing tablets but also placebo tablets prolonged the lifespan of peripherally prion-infected animals. Subsequent analysis of the tablet ingredients revealed that cellulose ethers (CEs) modified disease progression of the prion-infected animals. Here we report the anti-prion prophylactic efficacy of CEs, which have remarkable post- and pre-infection protective effects of a single injection into prion-infected animals. These compounds are non-digestible, non-ionic, water-soluble, polysaccharide derivatives commonly used as inactive ingredients in foods, cosmetics, and pharmaceuticals. We also examined the effectiveness of CEs when administered at various times post- or pre-infection in prion-infected animals. In addition, we examined the pharmacokinetics, biological features, structure-activity relationships, and effectiveness of CEs in other prion disease models, and mechanism of action. Finally the significance of the findings is discussed.

## Results

### Post-infection prophylactic effects

**Fig 1:**
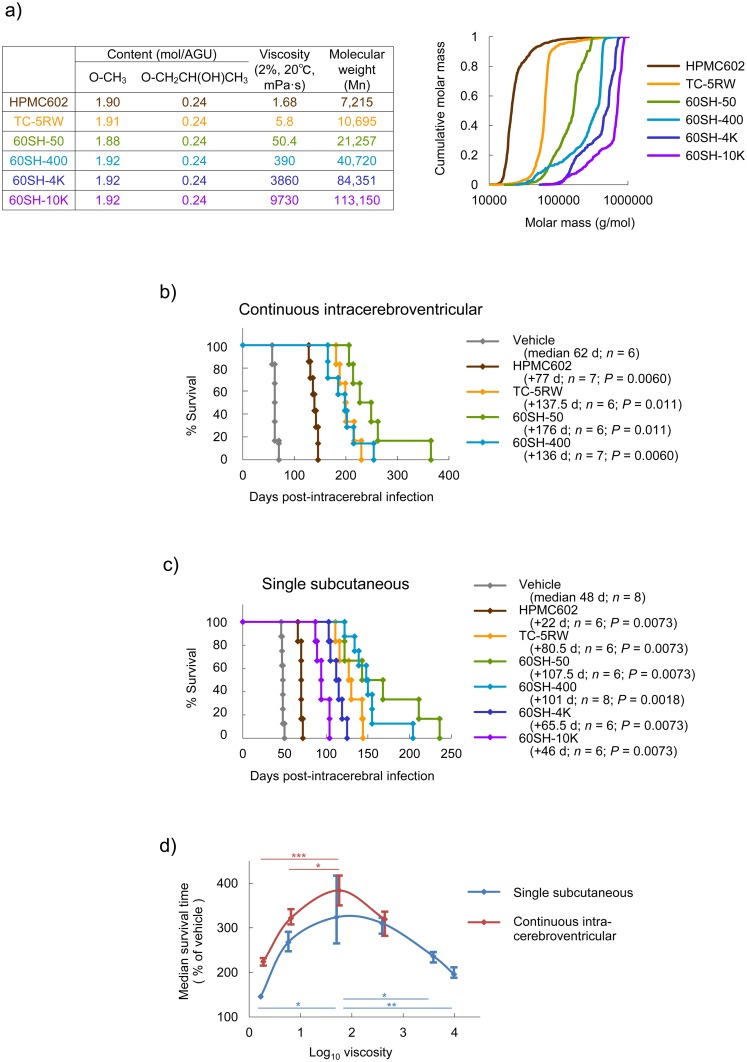
Post-infection prophylactic effects of CEs in intracerebral infection. (a) Chemical summary and cumulative molar mass of molecular weight distribution of HPMCs used in the study. Molecular weight distribution was analyzed by gel permeation chromatography (GPC) and dynamic light scattering of a 1% aqueous solution. AGU, anhydroglucose units. (b) Survival of Tg7 mice intracerebrally infected with the 263K prion and treated with HPMCs by a 4-week continuous intracerebroventricular infusion (150 μg/day) from 3 dpi. The log-rank test was used for survival analyses. (c) Survival of Tg7 mice intracerebrally infected with the 263K prion and treated with HPMCs by a single subcutaneous injection (4 g/kg body weight) at 0 dpi. The log-rank test was used for survival analyses. (d) Relationship between HPMC viscosity and survival time of animals treated post-infection. Medians and quartiles of the data of (b) and (c) are plotted. *P < 0.05; **P < 0.01; ***P < 0.005; log-rank test.

Hydroxypropyl methylcelluloses (HPMCs) were mainly used in this study because these compounds are the most popularly utilized CEs. First, we analyzed the post-infection effectiveness of HPMCs, which had similar contents of methyl modification [*ca*. 1.91 mol/anhydrous glucose unit (AGU)] and hydroxypropyl modification (0.24 mol/AGU), but different viscosities. Chemical summaries and cumulative molar masses of molecular weight distribution of HPMCs are shown in Fig 1a. It is obvious that depending on the viscosity, each HPMC had a distinct molecular weight distribution of polymers with certain ranges of molecular sizes.

Because it is expected that macromolecules are not delivered from the blood to the brain parenchyma through the blood–brain barrier, HPMCs were continuously infused into the cerebral ventricle to bypass the blood–brain barrier, as described in a previous study on pentosan polysulfate [12]. A 4-week continuous intracerebroventricular infusion (150 μg/day) of HPMC samples when given 3 days post-infection (dpi) demonstrated remarkable extension of survival times in Tg7 mice expressing hamster prion protein (PrP) [13–15] intracerebrally infected with the hamster-adapted 263K prion [16]. Although two of the most viscous HPMC samples could not be tested because of difficulty in handling the solution, the most effective was 60SH-50, which extended the median survival time by about 4-fold (62 days → 238 days), as compared to the vehicle control (Fig 1b).

On the other hand, unlike the case of previously reported macromolecules, such as pentosan polysulfate [12], it was interesting that a single subcutaneous injection (4 g/kg body weight) of HPMCs when given immediately after infection, demonstrated remarkable extension of survival times in Tg7 mice intracerebrally infected with the 263K prion. The most effective was also 60SH-50, which extended the median survival time by about 3-fold (48 days → 155.5 days), as compared to the vehicle control (Fig 1c).

When the median survival time (% of vehicle control) of HPMC-treated animals was plotted against HPMC viscosity, efficacy was obviously related to viscosity. The efficacy–viscosity relationships were bell-shaped, and an HPMC with a viscosity of approximately 100 mPa•s in a 2% solution at 20°C (equivalent to a 140-glucose-unit size) was most effective (Fig 1d). Meanwhile, CEs as short as hexamers were still effective when administered into the cerebral ventricle, although the effectiveness was not as remarkable as with large-sized CEs (S1 Fig).

The relationship between dosage and effectiveness revealed that efficacy was dependent on dosage by either single subcutaneous injection or 4-week continuous intracerebroventricular infusion (S2 Fig). Next, we determined whether variations in methyl or hydroxypropyl modification had any effect on the efficacy of CEs with similar viscosities and found that efficacy was not associated with the variations conferred by methyl or hydroxypropyl modifications (S3 Fig). In addition, we investigated whether contaminated materials had any effect on the efficacy of CEs and found that efficacy was not associated with contaminant removal by activated carbon treatment (S4 Fig). Although age [17] and sex [18,19] are known to affect the incubation times of prion-infected rodents at some instances, but the observed CE efficacy was similar in animals irrespective of these variables (S5 Fig).

### Post-infection prophylactic effects in peripheral infection

A single injection of a representative HPMC, TC-5RW, into either the peritoneal cavity or the tail vein demonstrated considerable efficacy in intracerebrally infected animals (S6a Fig). However, daily oral administration of TC-5RW to intracerebrally infected animals was not effective, even if it was started before they contracted the infection (S6b Fig). This result may be due to the poor intestinal absorption of CEs [20].

**Fig 2:**
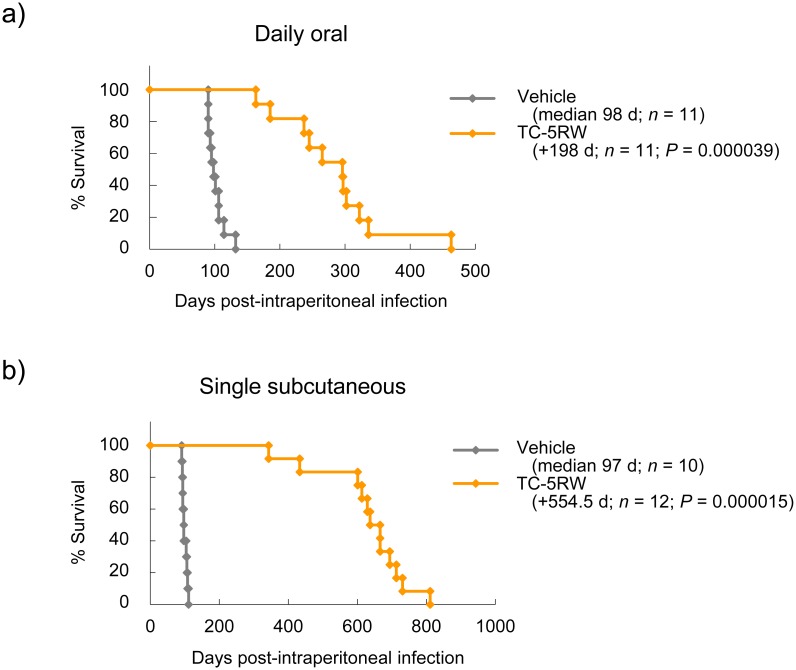
Prophylactic effects in intraperitoneal infection. (a) Survival of intraperitoneally prion-infected Tg7 mice treated with TC-5RW in a 15% TC-5RW-containing diet from 7 dpi to akinetic terminal disease. (b) Survival of intraperitoneally prion-infected Tg7 mice treated with TC-5RW by a single subcutaneous injection (4 g/kg body weight) at 3 dpi.

On the other hand, in the case of peripheral infection such as intraperitoneal infection, CE efficacy was enhanced, as compared to that of intracerebral infection. For instance, daily oral administration of TC-5RW, which was ineffective against intracerebral infection, was remarkably effective, as continuous treatment with a diet containing 15% TC-5RW from 7 dpi extended the median survival times of animals by about 3-fold (98 days → 296 days) (Fig 2a). A single subcutaneous injection of TC-5RW (4 g/kg body weight) at 3 dpi extended the median survival times of animals by about 6.7-fold (97 days → 651.5 days) (Fig 2b). The results suggest that even a small amount of CE absorbed through the intestinal tract is effective over a long period of time in case of peripheral infection, although absorption of CEs in the intestinal tract is reportedly very limited when orally administered [20].

### Post-symptomatic therapeutic effects

**Fig 3:**
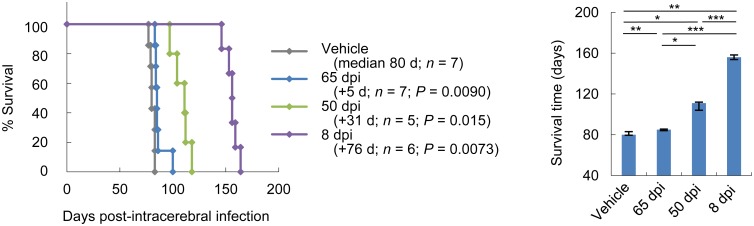
Post-symptomatic therapeutic effects. Survival analysis was performed in Syrian hamsters intracerebrally infected with the 263K prion and treated with a single subcutaneous injection (4 g/kg body weight) of TC-5RW at an early post-infection stage (8 dpi), a pre-symptomatic stage (50 dpi), or a post-symptomatic stage (65 dpi). A plot of medians and quartiles of the survival times is also shown. *P < 0.05; **P < 0.01; ***P < 0.005; log-rank test.

Because symptom onset in Tg7 mice was not clearly determined, Syrian hamsters were used. Syrian hamsters infected with the 263K prion showed ataxia or unstable gait as an initial obvious disease sign at approximately 60 dpi. Thus, we examined the effectiveness of TC-5RW given in pre-symptomatic or post-symptomatic disease stages. Similar to that of previously reported anti-prion materials [12,21], the efficacy of CEs decreased with the delay of post-infection timing of injection (Fig 3). A single administration of TC-5RW at an early symptomatic stage (65 dpi) although significantly effective, had a very limited effect on extending the survival.

### Pre-infection prophylactic effects

**Fig 4:**
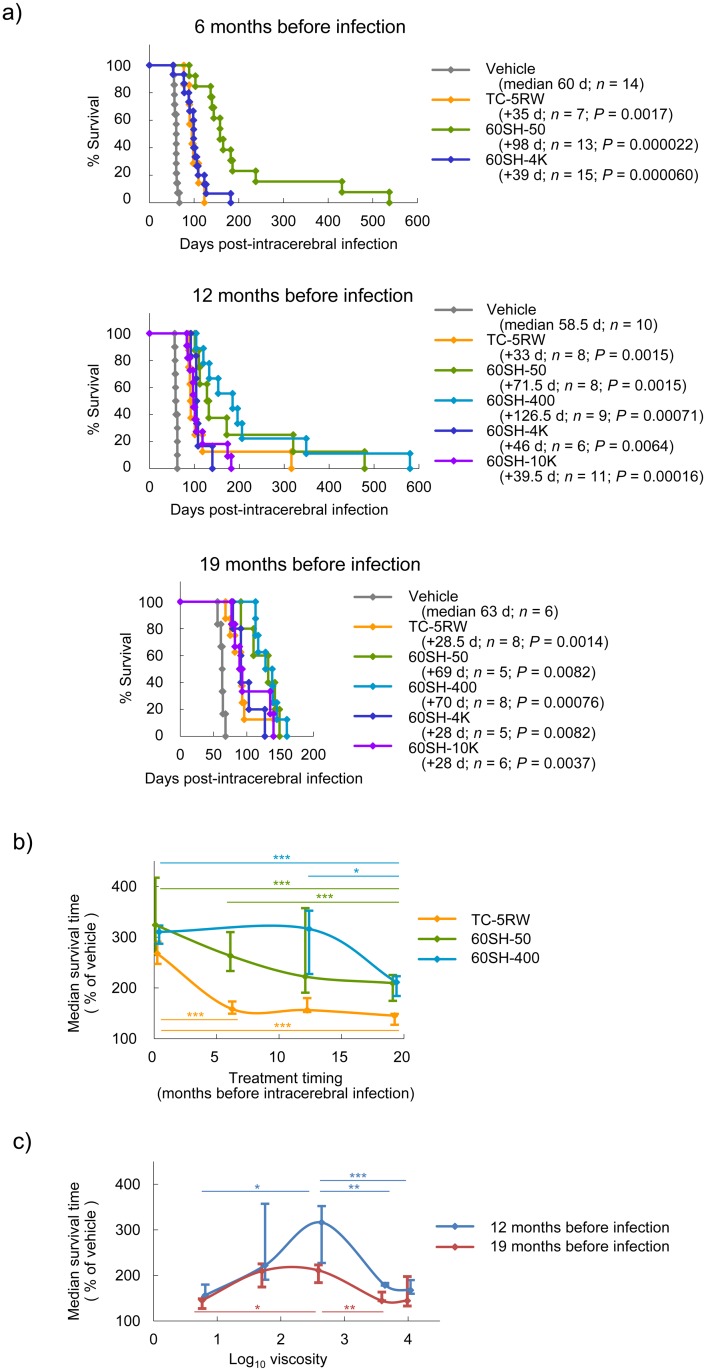
Pre-infection prophylactic effects in intracerebral infection. (a) Survival of intracerebrally 263K prion-infected Tg7 mice treated with HPMCs. A single subcutaneous injection (4 g/kg body weight) was given at 6, 12, or 19 months before infection. (b) Relationship between pretreatment timing and survival time of animals treated with HPMCs. Medians and quartiles from the data of representative HPMCs in (a) and Fig 1c are plotted. *P < 0.05; **P < 0.01; ***P < 0.005; log-rank test. (c) Relationship between HPMC viscosity and survival time of animals treated 12 or 19 months before infection. Medians and quartiles from the data of (a) are plotted. *P < 0.05; **P < 0.01; ***P < 0.005; log-rank test.

Because the effectiveness of a single post-infection CE administration continued over a long period, we examined the effectiveness of a single CE administration given months before infection and found that HPMCs given in a single subcutaneous injection prior to infection were still clearly effective. HPMCs given 6 or 12 months prior to infection were slightly less effective than, or as effective as, those given immediately after infection; even HPMCs given 19 months before infection were still markedly effective (Fig 4a).

When the median survival time (% of vehicle control) of HPMC-treated animals was plotted against the administration timing, efficacy tended to gradually decrease according to the pre-infection interval (Fig 4b). However, there was no significant difference in median survival times between the immediate post-infection intervention group and the 12-month pre-infection intervention group injected with 60SH-50 or 60SH-400. The most potent HPMC in the 12-month pre-infection intervention was slightly more viscous than the most potent HPMC in the post-infection intervention (Figs 1d and 4c).

Pre-infection prophylactic effects of CEs were also observed in Syrian hamsters (S7 Fig). By intraperitoneal infection, 60SH-50 or 60SH-400 extended the survival times of animals treated at the 0-month pre-infection by about 3.8-fold (233 days → about 890 days) and that of animals treated at the 13-month pre-infection by about 2.0- to 2.3-fold (195 days → 398 ~ 449 days). Therefore, it is obvious that even a single administration of CEs not only around the time of infection, but also as long as one year prior to infection, effectively extended the survival times of prion-infected animals.

### Pharmacokinetics of subcutaneously injected CE

To elucidate the mechanism of action of CEs, pharmacokinetics of subcutaneously injected radiolabeled TC-5RW were investigated in Tg7 mice. Immediately after injection, radiolabeled TC-5RW was taken up into the blood (S8a Fig) and excreted in the urine and feces at a rate of 70% of the initial dose within 3 days. Thereafter, it was excreted considerably more slowly, with 20% of the initial dose remaining in the body 2 weeks post-injection (S8b Fig). GPC analysis indicated that smaller-sized molecules of TC-5RW were preferentially excreted in the urine and feces, and larger-sized molecules remained in the plasma (S8c Fig).

**Fig 5:**
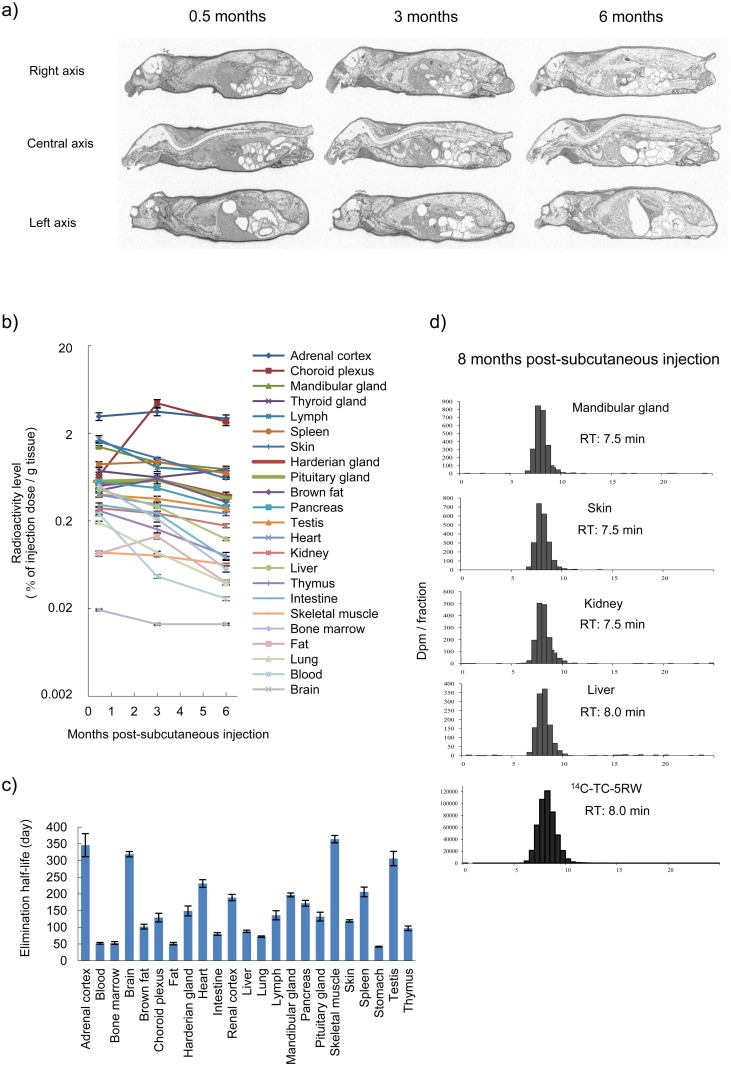
Long-term pharmacokinetics of subcutaneously injected ^14^C-TC-5RW. (a) Radioluminograms of Tg7 mice at 0.5, 3, or 6 months after a single subcutaneous injection of ^14^C-TC-5RW (2 g/kg body weight) (*n* = 1 for each time point). (b) Temporal profile of ^14^C-TC-5RW radioactivity levels in each tissue of Tg7 mice given a single subcutaneous ^14^C-TC-5RW injection. Radioactive signals in (a) were analyzed in technical triplicates, and the mean and standard deviation are presented. (c) Elimination half-life of ^14^C-TC-5RW in each tissue of Tg7 mice given a single subcutaneous ^14^C-TC-5RW injection. This half-life was calculated from the data presented in (b). (d) GPC profiles of ^14^C-TC-5RW radioactivity extracted from representative tissues of Tg7 mice 8 months after a single subcutaneous ^14^C-TC-5RW injection (2 g/kg body weight). The profile of ^14^C-TC-5RW used for the study is shown at the bottom.

Next, long-term pharmacokinetics and distribution were investigated in Tg7 mice given a single subcutaneous dose of radiolabeled TC-5RW. Radiolabeled TC-5RW was distributed in various tissues for long periods, but a little was found in the brain parenchyma, where prions accumulate and cause neurodegeneration (Fig 5a and 5b). Even after 6 months, amounts up to a few percent of the injected dose per gram of tissue or fluid were observed in various body parts, such as the adrenal cortex, choroid plexus, mandibular gland, thyroid gland, lymph, spleen, and skin, whereas less than 0.02 percent of the injected dose per gram of tissue (equivalent to ~10 μg/g tissue) was observed in the brain parenchyma. The elimination half-life of TC-5RW in these tissues was 50–350 days (Fig 5c) and longest elimination half-life periods were observed in the skeletal muscle, adrenal cortex, brain, and testis. GPC analysis revealed that larger portions of administered TC-5RW remained in tissues (Fig 5d). Consequently, a persistent residue of CE in the body is linked to long-lasting effects.

### Macrophage uptake analysis

**Fig 6:**
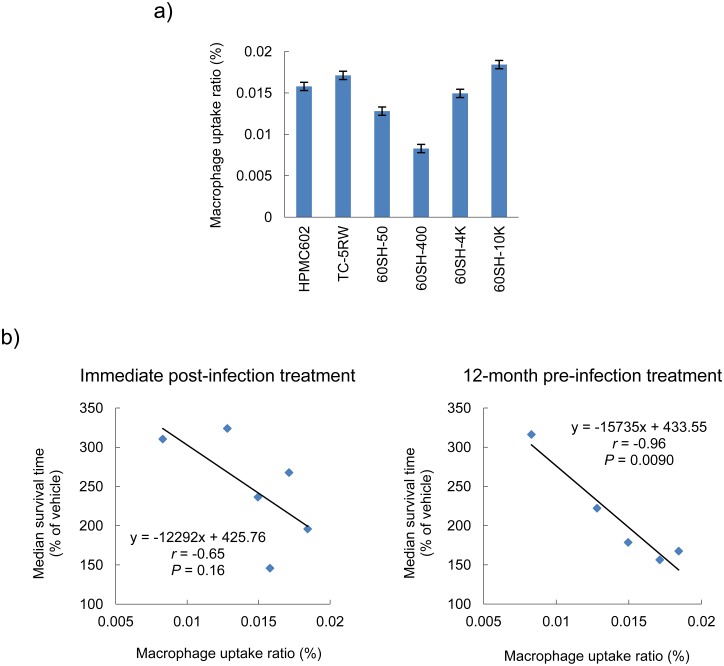
Macrophage uptake analysis. (a) HPMC macrophage uptake ratio. This represents the percentage of the HPMC amount of macrophage lysate *vs*. the amount added into the culture medium. The mean and standard deviation of triplicate experiments are shown. (b) Analysis of the linear correlation between the macrophage uptake ratio and prophylactic efficacy of HPMCs. Survival times of Tg7 mice treated with HPMCs immediately post-infection or treated 12 months prior to infection were obtained from Figs 1c and 4a. Statistical correlations were identified using the Pearson’s correlation coefficient.

Because CEs have been recognized to be biologically inactive macromolecules, we speculated that macrophages might be involved in the degradation and excretion of CEs administered in the body. Thus, we investigated macrophage uptake of CEs using fluorescein–labeled HPMCs and peritoneal macrophages of Tg7 mice and found that macrophage uptake ratios of HPMCs differed from each other and were independent of viscosity (Fig 6a). When compared with the data of the median survival times (% of vehicle control) of Tg7 mice treated with a single subcutaneous dose of HPMCs, the macrophage uptake ratio was inversely correlated, particularly with the survival time of 12-month pre-infection intervention, rather than that of the immediate post-infection intervention (Fig 6b). Therefore, more effective HPMCs in pre-infection protection were phagocytized to a lesser extent by macrophages.

### Structure–activity study

**Fig 7:**
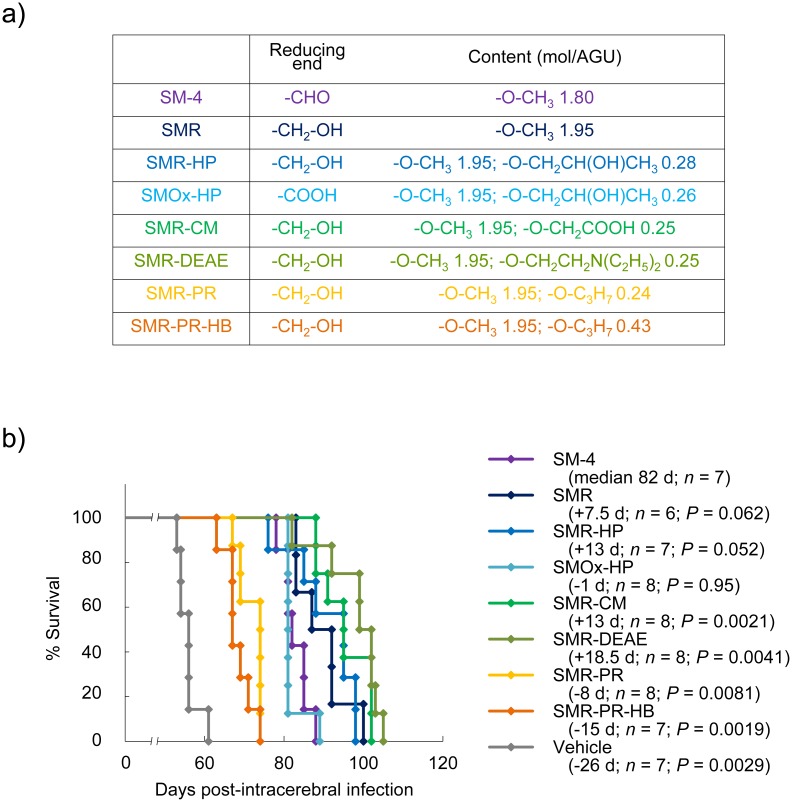
Structure–activity study of CEs. (a)Chemical summary of SM-4 derivatives tested in the study. (b) Survival of intracerebrally 263K prion-infected Tg7 mice treated with SM-4 derivatives. A single subcutaneous injection (2 g/kg body weight) was given at 1 dpi.

As a representative CE compound, methyl cellulose SM-4 was used for structure–activity studies. The chemical properties of tested compounds are shown in Fig 7a. The results of survival analysis of Tg7 mice treated with each compound are shown in Fig 7b. Comparison of SMR *vs*. SM-4 and SMR-HP *vs*. SMOx-HP indicated that a reduced modification of the reducing end produced better effects. Diethylaminoethyl modification (SMR-DEAE) produced the most excellent effects; carboxymethyl modification (SMR-CM) produced similar effects as hydroxypropyl modification (SMR-HP). In contrast to these hydrophilic modifications, hydrophobic modifications with a propyl group (SMR-PR and SMR-PR-HB) were much less effective than unmodified SMR. Further propyl group modification (SMR-PR-HB) was less effective than less modification (SMR-PR). These results suggest that the hydrophilicity of the CEs contributes to the effectiveness.

Hydroxypropyl and methyl ether compounds with sugar backbones other than cellulose were also analyzed in intracerebrally prion-infected Tg7 mice by a similar manner as performed with CEs. Tested sugar structures included dextran, dextrin, pullulan, starch, chitin, chitosan, and cyclodextrin. These polysaccharide ethers were ineffective (S1 Table), suggesting that the cellulose backbone is essential for effective protection against prion diseases.

### CE efficacies in other prion disease animal models

**Fig 8:**
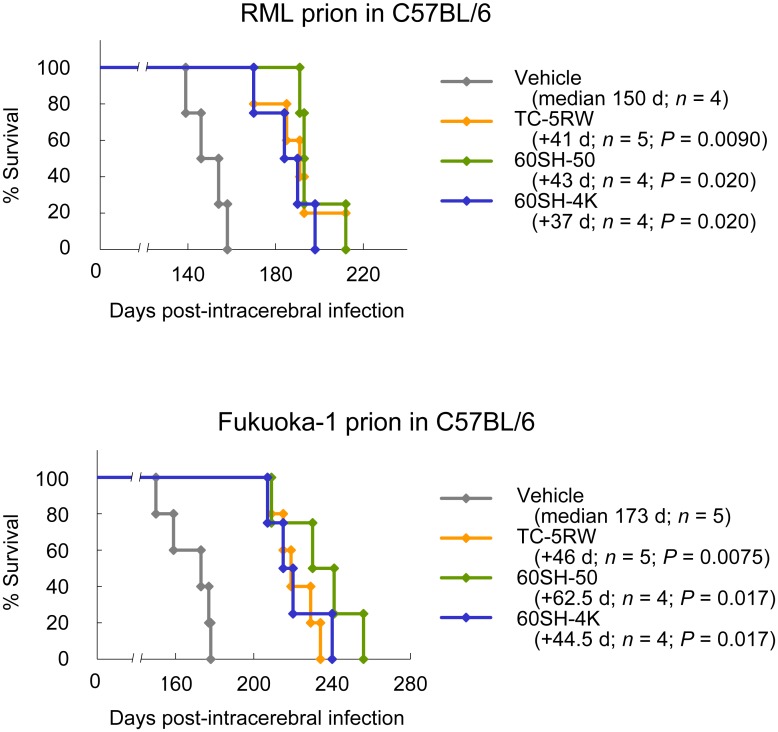
CE efficacies in other prion disease animal models. Survival analysis of C57BL/6 mice intracerebrally infected with the RML prion or Fukuoka-1 prion was performed. The mice were treated with a single subcutaneous injection of CEs at 4 g/kg body weight immediately after infection.

The effects of CEs were also investigated in C57BL/6 mice intracerebrally infected with mouse-adapted prion strains (RML scrapie prion and Fukuoka-1 human prion disease prion in Fig 8, and 22L scrapie prion in S9a Fig). The effects of CEs were significantly observed in all of the disease animal models, but were less remarkable than those observed in the 263K prion-infected Tg7 mice. Because it is inferable that animals with shorter incubation periods, such as Tg7 mice and Syrian hamsters, might show more remarkable CE effects than animals with longer incubation periods, we examined the effects of CEs in Tga20 mice, which have substantially shorter incubation periods to mouse-adapted prion strains [22]. The effects of CEs in Tga20 mice were significant but less remarkable (S9b Fig).

Therefore, prion disease animal models influenced the effects of CEs, and prion strain-associated factors or animal genetic backgrounds might contribute to the variation in CE efficacy. One example of the influence of the animal genetic background was demonstrated in the comparison between ddY and ICR mice. Both mice are outbred strains similarly used in drug toxicity and pharmacokinetic studies, but obvious differences in CE efficacy were observed between these mice (S10 Fig).

### Effects on PrP expression and conversion

**Fig 9:**
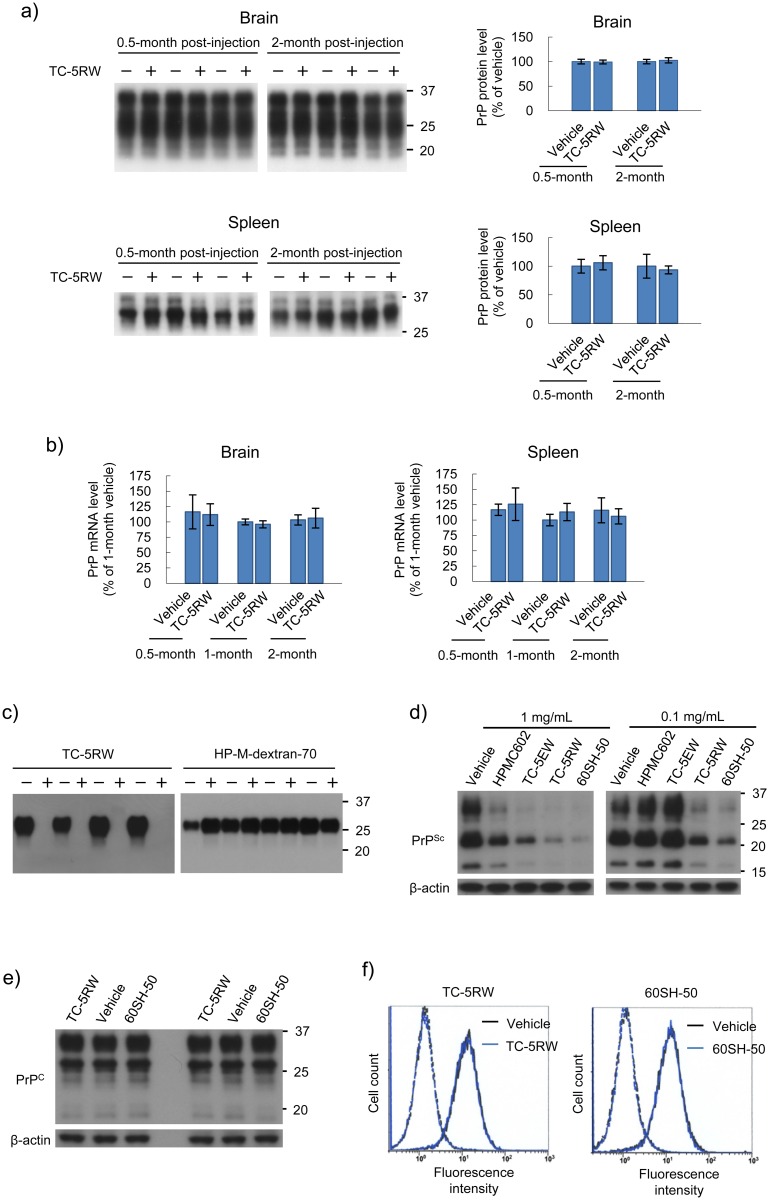
CE effects on PrP expression and PrP^Sc^ formation. (a) Immunoblotting of PrP^C^ in the brain and spleen from Tg7 mice treated with TC-5RW (4 g/kg body weight) or vehicle at the designated time points (*n* = 3 for each time point). Molecular size markers on the right indicate sizes in kDa. The mean and standard deviation are shown in each bar graph. (b) PrP mRNA expression level in the brain and spleen from Tg7 mice treated with TC-5RW (4 g/kg body weight) or vehicle at designated time points. The mean and standard deviation (*n* = 3 for each time point) are shown. (c) Immunoblotting of protease-resistant PrP^Sc^ in the protein misfolding cyclic amplification reaction performed in the presence of TC-5RW. Representative quadruplicate data are shown for the cyclic amplification of 263K prion performed in the presence (+) or absence (−) of TC-5RW or hydroxypropyl methyl dextran-70 kDa (HP-M-dextran-70) at a dose of 10 μg/mL. HP-M-dextran-70 was used as a negative control. In the absence of each compound, the vehicle, water, was added to the reaction mixture. (d) Immunoblotting of protease-resistant PrP^Sc^ in RML prion-infected N2a cells treated with HPMCs. Signals for β-actin are shown as controls for the integrity of samples used for PrP^Sc^ detection. The chemical property and *in vivo* efficacy of TC-5EW are shown in S4 Fig. (e) Immunoblotting of PrP^C^ in uninfected N2a cells treated with HPMCs at a dose of 1 mg/mL in duplicate. (f) Flow cytometry of cell surface PrP^C^ in uninfected N2a cells treated with HPMCs at a dose of 1 mg/mL. Black and blue lines indicate vehicle-treated cells and HPMC-treated cells, respectively. The broken line peaks on the left show the respective isotype controls.

In prion diseases, normal PrP (PrP^C^) is conformationally converted to an abnormal protease-resistant PrP (PrP^Sc^) as the main component of the prion [1]. However, administration of CEs did not modify the protein or gene expression level of PrP in the brain or spleen (Fig 9a and 9b). On the other hand, administration of CEs inhibited 263K prion amplification in the protein misfolding cyclic amplification reaction, which is a method to amplify PrP^Sc^ in vitro [23], whereas a control compound, hydroxypropyl methyl dextran (70 kDa), did not inhibit amplification (Fig 9c). Similarly, in persistently RML prion-infected cells [24], CEs inhibited PrP^Sc^ formation in accordance with their in-vivo potencies, although higher concentrations of CEs were needed (Fig 9d). CE administration did not affect the expression levels of total PrP^C^ or cell surface PrP^C^ of the cells (Fig 9e and 9f). These results suggest that CEs inhibit PrP^C^–PrP^Sc^ conversion without affecting PrP^C^ metabolism.

## Discussion

### CEs as new anti-prion compounds

The results of the present study revealed that CEs, as new types of anti-prion compounds, can effectively extend the life span of animals infected with prion not only peripherally but also intracerebrally, even though CEs are macromolecules that are hardly capable of reaching the brain parenchyma. CEs share no similarities in their structures or chemical properties with previously reported anti-prion polymers, such as sulfated glycans [25,26], polyamines [27–31], and cationic dendrimers [32–35]. Unlike these anionic and cationic polymers, CEs are non-ionic but hydrophilic.

The long-lasting efficacy of CEs is an astonishing, distinct feature from any other compounds previously reported [3–6,36–38]; a single post-infection subcutaneous injection of CEs extended the life spans of peripherally infected mice nearly to natural life spans and extended the life spans of intracerebrally infected mice by 2–3 fold. More astonishing is the pre-exposure prophylactic effects of CEs, as no significant difference in efficacy was observed in the most effective CE administered at an immediate post-infection or at one year pre-infection. No other compounds or biological materials exogenously administered have been reported to achieve these levels of post-infection and pre-infection prophylactic effects in peripherally or intracerebrally prion-infected animals.

### Biological and chemical properties of effective CEs

CE molecules were distributed throughout the body within days after a single subcutaneous injection, and larger-sized portions of the molecules were retained in the body for months. The ability of CE residues to remain in the body is associated with the long-lasting protective effects of CEs. Chemically, the long-lasting protective efficacies of CEs were associated with the molecular size, but not the degree of modification to the methyl or hydroxypropyl group. Biologically, long-lasting protective efficacies were significantly associated with macrophage uptake ratios: CEs less phagocytized by macrophages were more effective. These data appear to be consistent with the structure-activity data, which shown that the efficacies of CEs were abolished by hydrophobic, but not hydrophilic, modifications. This can be explained by the findings of Tabata and Ikada who reported that microspheres with hydrophobic surfaces are more readily phagocytized than those with hydrophilic surfaces [39]. Therefore, it is presumable that macrophages play a crucial role in the efficacies of CEs and that phagocytosis facilitates the decomposition or excretion of CEs.

### Mechanism of action of CEs

CEs are unlikely to affect the metabolism or turnover of PrP^C^ because the expression levels of PrP^C^ were not modified in tissues of TC-5RW-treated mice or TC-5RW-treated culture cells. However, CEs might directly inhibit the conversion of PrP^C^ to PrP^Sc^ (namely prion formation) in prion-infected animals because of the following findings: TC-5RW inhibited prion formation in the protein misfolding cyclic amplification reaction; CEs were remarkably effective when infused into the cerebroventricular system; and radioactivity of TC-5RW was observed in the brain parenchyma at an amount of less than 10 μg/g tissue equivalent and was eliminated very slowly in the brain parenchyma at a half-life of about one year.

On the other hand, there was a gap in the CE concentration required for inhibition of prion formation among the assay systems. CE concentration of less than 10 μg/g tissue equivalent was needed in the mouse brain, and this concentration appeared to be similar to that of the protein misfolding cyclic amplification reaction (~10 μg/mL), whereas it was different from that of the prion-infected cells (~1 mg/mL). Because the prion strain differed among these assay systems, the CE concentration gap might reflect the prion strain dependency of CE action: significantly effective against the 263K prion, but much less effective against the RML prion. However, it is possible that the mechanism of action of CEs differs among the assay systems, and the possibility cannot be ruled out that other factors, such as CE-metabolites or CE-induced host factors, are responsible for the anti-prion activities of CEs in animals.

### CE efficacy is influenced by prion disease animal models

Although CE effects were significantly observed in all prion disease animal models tested in the present study (except for the ICR mouse model), CE effects in animals infected with mouse-adapted prions were not as remarkable as those of animals infected with the 263K hamster-adapted prion. In addition, CE effects were not necessarily remarkable in animal models with shorter incubation periods. Thus, these findings imply that CE effects may be influenced by the prion strain or animal genetic background.

Because no animals with an identical genetic background are available for comparison of CE efficacies against hamster-adapted prions *vs*. mouse-adapted prions, it is not possible at the present time to verify the influence associated with prion strains. Regarding the animal genetic background, Tg7 mice have a mixed background with 129/Ola and C57BL/10 [13–15]. Thus, the genetic background of Tg7 mice is very different from that of C57BL/6, whereas Tga20 mice have a genetic background close to C57BL/6 [22]. Accordingly, the animal genetic background might influence the effects of CEs. Although this inference remains to be elucidated, the data of the comparison between ddY and ICR mice are suggestive of inference.

### CEs as a preventive drug model

Tremendous efforts have been made to conduct clinical trials or experimental treatments of several compounds [40–45]. However, there have been no meaningful outcomes in regard to patient benefit. One of the reasons clinical trials commonly fail in terms of survival is supposed to be delayed intervention. In prion-infected animals, the larger delay in intervention, the less effective anti-prion compounds are at prolonging survival [12,21]. The most opportune time for therapeutic intervention of prion diseases is very early in the preclinical stage because exponentially accumulated amounts of the prion almost reach a plateau in the brain around the stage of symptomatic disease [46,47].

Because the effectiveness of CEs administered after disease onset were also very limited, despite being statistically significant, therapeutic effects are hardly expected for intervention after disease onset. However, significantly beneficial effectiveness of CEs might be expected by immediate post-infection or pre-exposure prophylactic intervention. Especially at peripheral infection, a few or several injections of CEs might be sufficiently preventive to keep infected individuals healthy for the expected lifetime. However, the safety properties of CEs remain to be evaluated and drug availability to target tissues must be improved before the dosing regimen can be optimized.

In conclusion, it is not yet clear how polymers, such as CEs, peripherally administered suppress the disease process in the brain and extend survival. It is also unclear how CE efficacy is influenced by prion strains or host genetic factors. These enigmas await further elucidation. However, the findings of this study suggest that CEs may be something in daily life that modify disease onset and could be useful for the development of preventive measures against prion diseases.

## Materials and Methods

### Ethics statement

All animal experiments were performed in accordance with protocols reviewed and approved by the Institutional Animal Care and Use Committee of Tohoku University (approval numbers 20MdA-54, 21MdA-192, 22MdA-247, 2011MdA-347, 2012MdA-272, 2013MdA-194, and 2016MdA-139). The animal care and use protocols adhered to the Fundamental Guidelines for Proper Conduct of Animal Experiment and Related Activities in Academic Research Institutions by the Ministry of Education, Culture, Sports, Science and Technology (Notice No. 71 issued on June 1, 2006), the Standards Relating to the Care and Management of Laboratory Animals and Relief of Pain by the Ministry of the Environment (Notice No. 84 issued on August 30, 2013), and the Act on Welfare and Management of Animals (revised on September 5, 2012) in Japan.

### CEs

All HPMC compounds were kindly provided by Shin-Etsu Chemical Co., Ltd. (Tokyo, Japan). Compound properties are listed in Fig 1a. Methyl cellulose (SM-4) was also provided by Shin-Etsu Chemical Co., Ltd. All SM-4 derivatives were synthesized from SM-4 by Meito Sangyo Co., Ltd. (Nagoya, Japan). A chemical summary of SM-4 and its derivatives is presented in Fig 7a.

### Animal study

Tg7 mice, kindly provided by Dr. Bruce Chesebro of the Laboratory of Persistent Viral Diseases of NIAID’s Rocky Mountain Laboratories (Hamilton, MT, USA), were mainly used in the study because they have substantially shorter incubation times than Syrian hamsters when intracerebrally infected with the hamster-adapted 263K scrapie prion [13–15]. Tg7 mice are derived from transgenic Tg10 mice [13] and are crossed onto a mouse PrP-null background [48]. Thus, Tg7 mice lack endogenous mouse PrP^C^ expression but express hamster PrP^C^ at about 4 times the level of that expressed by wild-type mice in the brain and at a level similar to that expressed by wild-type mice in the spleen (S11 Fig). Six- to 10-week-old male mice were used for all experiments, unless otherwise stated. Intracerebral or intraperitoneal infection was performed by inoculation with 20 or 100 μL, respectively, of a 1% (w/v) brain homogenate obtained from a terminally ill 263K prion-infected hamster. In case of continuous intracerebroventricular infusion, continuous infusion into the cerebral third ventricle was performed using an osmotic minipump and brain infusion kit, as previously described [49]. Mice were monitored every day until the time of terminal disease, at which time the mice were akinetic (with a lack of grooming behavior, coordination, and parachute reaction) or exhibited a rigid tail, an arched back, and weight loss of approximately 10% within one week. The mice were killed at this stage and disease was confirmed by immunoblotting and immunohistochemical analyses of abnormal PrP deposition in the brain, as previously described [12,49]. Because it was difficult to determine the time of symptom onset in the animal model used in this study, survival time was defined as the time from prion infection to terminal disease.

Experiments with Syrian hamsters and other mice were performed in a similar manner, as described above. Syrian hamsters and C57BL/6 mice were purchased from Japan SLC, Inc. (Hamamatsu, Japan). In the case of post-infection CE administration into intraperitoneally prion-infected hamsters, hamsters in all experimental groups were given a single subcutaneous injection of CEs in such a manner that all intraperitoneal prion infections were performed with animals of the same age.

### Pharmacokinetics of ^14^C-TC-5RW

^14^C-TC-5RW was synthesized by the condensation of NaOH-treated TC-5RW with ^14^C-methyl iodide, and a pharmacokinetic study of ^14^C-TC-5RW in Tg7 mice was performed at Sekisui Medical Co., Ltd. (Tsukuba, Japan) as follows. A designated amount of ^14^C-TC-5RW in saline was subcutaneously injected into the backs of mice. After a designated interval, mice under deep anesthesia were frozen and embedded in 4% carboxymethyl cellulose-Na. Whole-body 30-μm-thick sections were cut with a cryomicrotome and dried. Sections accompanied by radioactive standards were exposed to imaging plates and radioluminograms were analyzed with a BAS2500 bio-imaging analyzer (Fuji Film, Tokyo, Japan). Considering both the standard calibration curve data and the ^14^C-TC-5RW specific radioactivity, radioactivity levels in each tissue or fluid sample were calculated from the radioluminograms. Elimination half-life was calculated using the following formula: elimination half-life (days) = –ln_2_/β, where β is the slope obtained from the simple linear regression of the natural logarithm of radioactivity levels on post-injection days. To measure the molecular sizes of ^14^C-TC-5RW residues in tissues, tissue samples were homogenized with five volumes of 0.1 N NaOH on ice, and the supernatants after centrifugation at 12,000×g were subjected to GPC analysis. Samples were separated on an OHpak SB-804 HQ column (Showa Denko, Tokyo, Japan) and eluted with 0.1 M NaCl at 1 mL/min and 35°C. Each 0.5-min fraction was mixed with the scintillation cocktail and radioactivity was assayed by liquid scintillation counting.

### Macrophage uptake analysis

Fluorescein-labeled HPMCs were produced as follows. HPMC in dimethylformamide (0.3 g/30 mL) was mixed with fluorescein-5-carbonyl azide diacetate (1.5 mg). The mixture was purged with N_2_ gas and agitated at 90°C for 3 h. Labeled HPMC was recovered and then purified using repeated precipitation–solubilization steps. Residual solids were dissolved in NaHCO_3_ solution (100 mM, pH 8.0) at room temperature for 8 h to remove the acetyl-protecting groups and subsequently dialyzed against deionized water. The solution was filtered through a 0.45-μm pore size filter (EMD Millipore, Billerica, MA, USA) and then lyophilized. Fluorescein-labeled HPMCs showed no fluorescence signals in the low-molecular-weight fractions on a GPC instrument equipped with a fluorescence detector (column, OHpak SB-804 HQ; detection, excitation at 494 nm and recording at 520 nm). Peritoneal macrophages were obtained from Tg7 mice by conventional thioglycollate induction. Cells (3 × 10^5^) were incubated with fluorescein-labeled HPMC (1 mg/mL) in culture medium for 1 day and then rinsed twice and lysed in a lysis buffer (0.5% sodium deoxycholate, 0.5% Nonidet P-40, phosphate-buffered saline (PBS), pH 7.4). The amount of fluorescein-labeled HPMC in the cell lysate was determined by GPC analysis.

### PrP expression analysis

Brains were homogenized in two volumes of TN buffer (50 mM Tris HCl, 0.1 M NaCl, pH 7.5) supplemented with a protease inhibitor cocktail. Spleens were homogenized and sonicated in 13 volumes of TN buffer supplemented with 0.5% sodium deoxycholate, 0.5% Nonidet P-40, and the protease inhibitor cocktail. After low-speed centrifugation, aliquots of supernatant containing the same protein amount were analyzed for PrP^C^ protein levels by immunoblotting with the anti-PrP monoclonal antibody 3F4 (BioLegend, Inc., San Diego, CA, USA), as previously described [19]. To analyze PrP mRNA expression, the brains and spleens of Tg7 mice were lysed using an RNA extraction reagent. Total RNA was extracted and cDNA was synthesized using a first-strand cDNA synthesis kit (Takara Bio, Inc., Kyoto, Japan). The PrP mRNA level was measured by real-time polymerase chain reaction, as previously described [49].

### Protein misfolding cyclic amplification

According to a previously described method [50], a reaction mixture containing 0.1% 263K prion-infected hamster brain homogenate and 10% normal hamster brain homogenate underwent 96 cycles of sonication and incubation with an automatic cross-ultrasonic apparatus (ELESTEIN 070-GOTW; Elekon Science Corp., Chiba, Japan). TC-5RW or hydroxypropyl methyl dextran (70 kDa) was added to the reaction mixture before starting the reactions at concentrations ranging 10 μg/mL to 1 mg/mL. After the reactions, the samples were digested with 100 μg/mL of proteinase K at 37°C for 1 h and levels of protease-resistant PrP^Sc^ were detected by immunoblotting with 3F4.

### Analysis in prion-infected cells

Mouse neuroblastoma cells either uninfected (N2a cells) or persistently infected with the RML prion (ScN2a cells [24]) were kindly provided by Dr. Byron Caughey of the Laboratory of Persistent Viral Diseases of NIAID’s Rocky Mountain Laboratories (Hamilton, MT, USA) and used as previously described [21,51,52]. Briefly, cells were cultured in the presence of test compounds for 3 days. Cells grown to confluency were washed with PBS and lysed with lysis buffer (0.5% sodium deoxycholate, 0.5% Nonidet P-40, PBS, pH 7.4). For the detection of PrP^Sc^, cell lysate was treated with 10 μg/mL of proteinase K at 37°C for 30 min and subsequently with 1 mM phenylmethylsulfonyl fluoride. Then, PrP^Sc^ were precipitated by centrifugation at 10,000 × *g* and suspended in a sample loading buffer. To detect PrP^C^ and β-actin, cell lysate was used without further treatments and mixed with a concentrated loading buffer. Immunoblotting analysis was performed using the anti-PrP monoclonal antibody SAF83 (SPI-Bio, Massy, France) and anti-β-actin monoclonal antibody, as described previously [49]

The cell surface of PrP^C^ was analyzed by flow cytometry, as described in previous reports [21,49,52]. Briefly, N2a cells incubated in the presence of test compounds for 3 days were dispersed using 0.1% collagenase and washed with ice-cold 0.5% fetal calf serum in PBS. Then, the cells were immunoreacted with SAF83 or isotype control IgG1 for 30 min on ice, and subsequently with goat F(ab’)2 fragment anti-mouse IgG (H+L)-FITC (Beckman Coulter, Inc., Brea, CA, USA) for 30 min on ice. Then, the cells were analyzed using an EPICS XL-ADC flow cytometer (Beckman Coulter, Inc.).

### Statistical analysis

Survival rates were calculated using the Kaplan–Meier method and significance was evaluated using the log-rank method. Statistical linear correlations were evaluated by the Pearson’s correlation coefficient. All analyses were performed using Excel Toukei, a statistical analysis software (SSRI Co., Ltd., Tokyo, Japan).

## Supporting Information

S1 Fig Efficacy of low-molecular-size CEs. Survival analysis of Tg7 mice intracerebrally infected with the 263K prion and treated with hexamer CEs via a 4-week continuous intracerebroventricular infusion (150 μg/day) from 2 dpi was performed. Hexamer CEs were synthesized from hexacellulose by Meito Sangyo Co., Ltd. (Nagoya, Japan). M-6Cr had an O-CH_3_ content of 1.81 mol/AGU and a reduced OH terminus, whereas HPM-6Cr had an O-CH_2_CH(OH)CH_3_ content of 0.27 mol/AGU in addition to M-6Cr chemical properties.
(TIF)

S2 Fig Dose response of TC-5RW. TC-5RW dose response was analyzed in Tg7 mice intracerebrally infected with the 263K prion via a single subcutaneous injection at 0 or 7 dpi or a 4-week continuous intracerebroventricular infusion from 8 dpi. Median and quartiles are shown (*n* = 5 or 6 for each time point).
(TIF)

S3 Fig Effect of changes in CE modification. Survival analysis was performed in Tg7 mice intracerebrally infected with the 263K prion and treated 3 days pre-infection with a single subcutaneous injection (2 g/kg body weight) of HPMCs with similar viscosities but different modifications (60SH-4K *vs*. 90SH-4K and HPMC602 *vs*. HPMC902).
(TIF)

S4 Fig Effect of activated carbon treatment on CE efficacy. Survival analysis was performed as described in S3 Fig using either activated carbon-treated HPMCs or untreated HPMCs (TC-5RW *vs*. TC-5R and TC-5EW *vs*. TC-5E).
(TIF)

S5 Fig CE efficacy independent of age and sex of animals. Box plots of survival periods in old (43–53 months) and young (6–10 months) male (M) and female (F) mice are shown. Tg7 mice were treated with a single intraperitoneal injection (1 g/kg body weight) 1 day before intracerebral infection with the 263K prion. No significant difference was observed among TC-5RW-treated groups (*n* = 5 or 6 for each group) or vehicle-treated groups (*n* = 6 for each group); log-rank test.
(TIF)

S6 Fig Prophylactic effects of CEs via other administration routes. (a) Survival analysis was performed in Tg7 mice intracerebrally infected with the 263K prion and treated with TC-5RW via a single intraperitoneal infusion (1 g/kg body weight) immediately after infection or via a single intravenous injection (0.3 g/kg body weight) 6 h pre-infection. (b) Survival analysis was performed in Tg7 mice intracerebrally infected with the 263K prion and treated with a 15% TC-5RW-containing diet from 9 days pre-infection to akinetic terminal disease.
(TIF)

S7 Fig Pretreatment efficacy in intraperitoneally infected hamsters. Survival analysis was performed in Syrian hamsters intraperitoneally infected with the 263K prion and treated with HPMCs via a single subcutaneous injection (4 g/kg body weight) at 0 or 13 months before infection.
(TIF)

S8 Fig Pharmacokinetics of subcutaneously injected ^14^C-TC-5RW; absorption and excretion. (a) Blood ^14^C-TC-5RW radioactivity levels in Tg7 mice that received a single subcutaneous injection of ^14^C-TC-5RW at 2 g/kg body weight. The mean and standard deviation are shown from technical triplicates at 3 or 6 months (*n* = 1) and from biological triplicates at other time points (*n* = 3). (b) Kinetics of ^14^C-TC-5RW radioactivity excretion by Tg7 mice that received a single subcutaneous injection of ^14^C-TC-5RW at 0.1 g/kg body weight. Total excretion radioactivity levels were determined from whole-body residual radioactivity levels. The mean and standard deviation are shown (*n* = 3). (c) ^14^C-TC-5RW molecular size profiles in the plasma, urine, and feces of Tg7 mice described in (b). GPC profiles of radioactivity were analyzed in plasma and excreta within 2 weeks and 3 days, respectively, of post-subcutaneous injection of ^14^C-TC-5RW (*n* = 1). RT, peak retention time; psi, post-subcutaneous injection. Methods: A designated amount of ^14^C-TC-5RW in saline was subcutaneously injected into the backs of mice. Blood or excreta samples were collected from the mice at designated time points. Urine and feces were collected in metabolic cages and expired CO_2_ was captured in 20% ethanolamine solution. Feces were dissolved with a scintillation solubilizer. The whole body was solubilized in heated 0.5 N NaOH/5% toluene solution to assay whole-body residual radioactivity. Portions of samples were mixed with a scintillation cocktail and radioactivity was assayed by liquid scintillation counting. GPC analysis was performed in a similar manner as described in the Materials and Methods section.
(TIF)

S9 Fig CE efficacies in other prion disease animal models. (a) Survival analysis of C57BL/6 mice intracerebrally infected with the 22L prion and treated with a single subcutaneous injection of CEs at 2 g/kg body weight 1 day before infection.(b) Survival analysis of Tga20 mice intracerebrally infected with the RML prion and treated with a single subcutaneous injection of CEs at 4 g/kg body weight immediately after infection. Tga20 mice overexpressing mouse PrP^C^ [22] were kindly provided by Dr. Charles Weissmann of the Scripps Research Institute (La Jolla, CA, USA).
(TIF)

S10 Fig Comparison of CE efficacy in two mouse strains. Survival analysis of ddY and ICR mice intracerebrally infected with the RML prion and treated with a single subcutaneous injection of TC-5RW (2.5 g/kg body weight) 1 day before infection. These mice were purchased from Japan SLC, Inc. (Hamamatsu, Japan).
(TIF)

S11 Fig PrP^C^ expression in Tg7 mice. (a) PrP^C^ levels in the brain from Tg7 and C57BL/6 (B6) mice. Brain homogenates containing 5 μg protein were analyzed by immunoblotting with SAF83 antibody (*n* = 4 for each mouse strain). Molecular size markers on the right indicate sizes in kDa. (b) Quantitative comparison of PrP^C^ levels in the brain between Tg7 and B6 mice. Two of the Tg7 brain samples in (a) were diluted and the PrP^C^ levels were compared with those of undiluted B6 brain samples by immunoblotting. A plot of relative signal intensities for Tg7 samples (% of B6) is shown against dilution ratios; the mean and standard deviation were obtained from technical triplicate analysis (*n* = 2 for each mouse strain). (c) PrP^C^ levels in the spleen from Tg7 and B6 mice. Spleen homogenates containing 10 μg protein were analyzed by immunoblotting as described in (a) (*n* = 4 for each mouse strain). The mean and standard deviation of signal intensities are shown. NS not significant; t-test.
(TIF)

S1 Table Other tested polysaccharide ethers. Survival analyses were performed in 263K prion-infected Tg7 mice treated with a single dose of each sample at a designated timing, as described in the Materials and Methods section. Dextran and dextrin were provided by Meito Sangyo (Nagoya, Japan) and pullulan by Hayashibara (Okayama, Japan). Hydroxypropyl methyl ethers of these compounds were synthesized by Meito Sangyo. Other compounds were purchased from Sigma-Aldrich, Tokyo Chemical Industry, and Wako Pure Chemicals (Tokyo, Japan).
(DOCX)
