# Supplementary figures and images for: A Single Subcutaneous Injection of Cellulose Ethers Administered Long before Infection Confers Sustained Protection against Prion Diseases in Rodents
Source: PLoS Pathog. 2016 Dec 14;12(12):e1006045. doi: 10.1371/journal.ppat.1006045 (PMC5156379; doi:10.1371/journal.ppat.1006045)

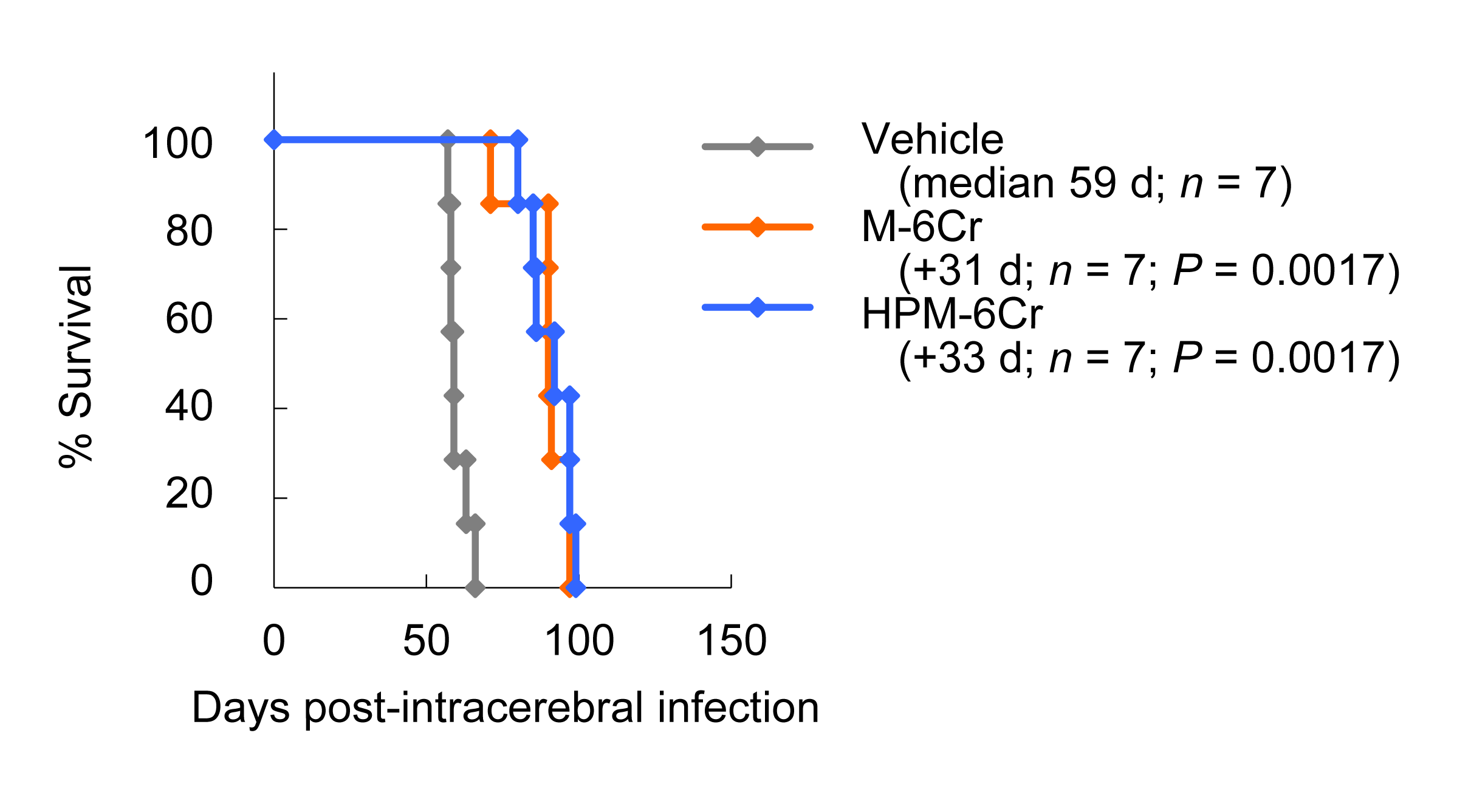

Supplement: S1 Fig — Survival analysis of Tg7 mice intracerebrally infected with the 263K prion and treated with hexamer CEs via a 4-week continuous intracerebroventricular infusion (150 μg/day) from 2 dpi was performed. Hexamer CEs were synthesized from hexacellulose by Meito Sangyo Co., Ltd. (Nagoya, Japan). M-6Cr had an O-CH3 content of 1.81 mol/AGU and a reduced OH terminus, whereas HPM-6Cr had an O-CH2CH(OH)CH3 content of 0.27 mol/AGU in addition to M-6Cr chemical properties. (TIF) [file ppat.1006045.s001.tif]

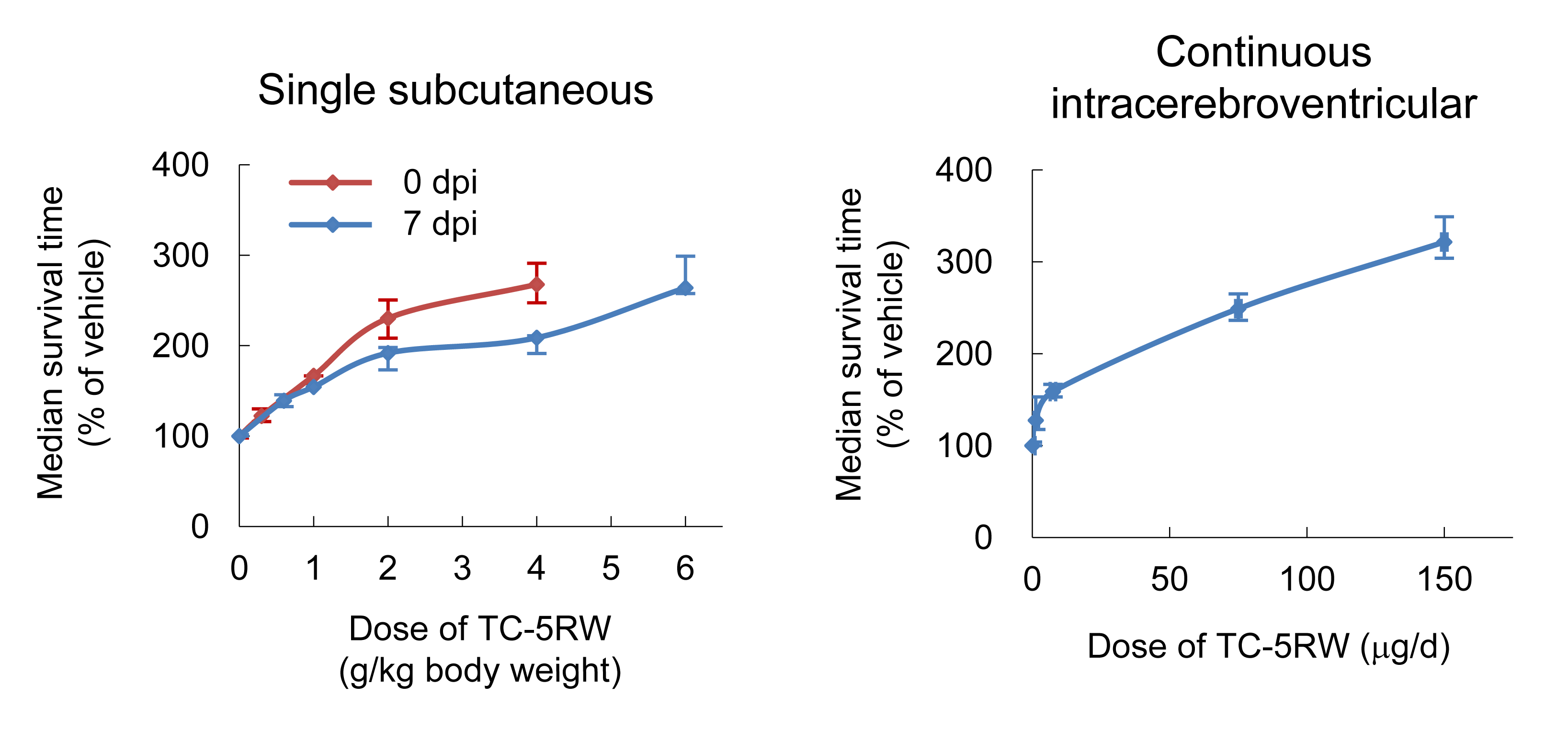

Supplement: S2 Fig — TC-5RW dose response was analyzed in Tg7 mice intracerebrally infected with the 263K prion via a single subcutaneous injection at 0 or 7 dpi or a 4-week continuous intracerebroventricular infusion from 8 dpi. Median and quartiles are shown (n = 5 or 6 for each time point). (TIF) [file ppat.1006045.s002.tif]

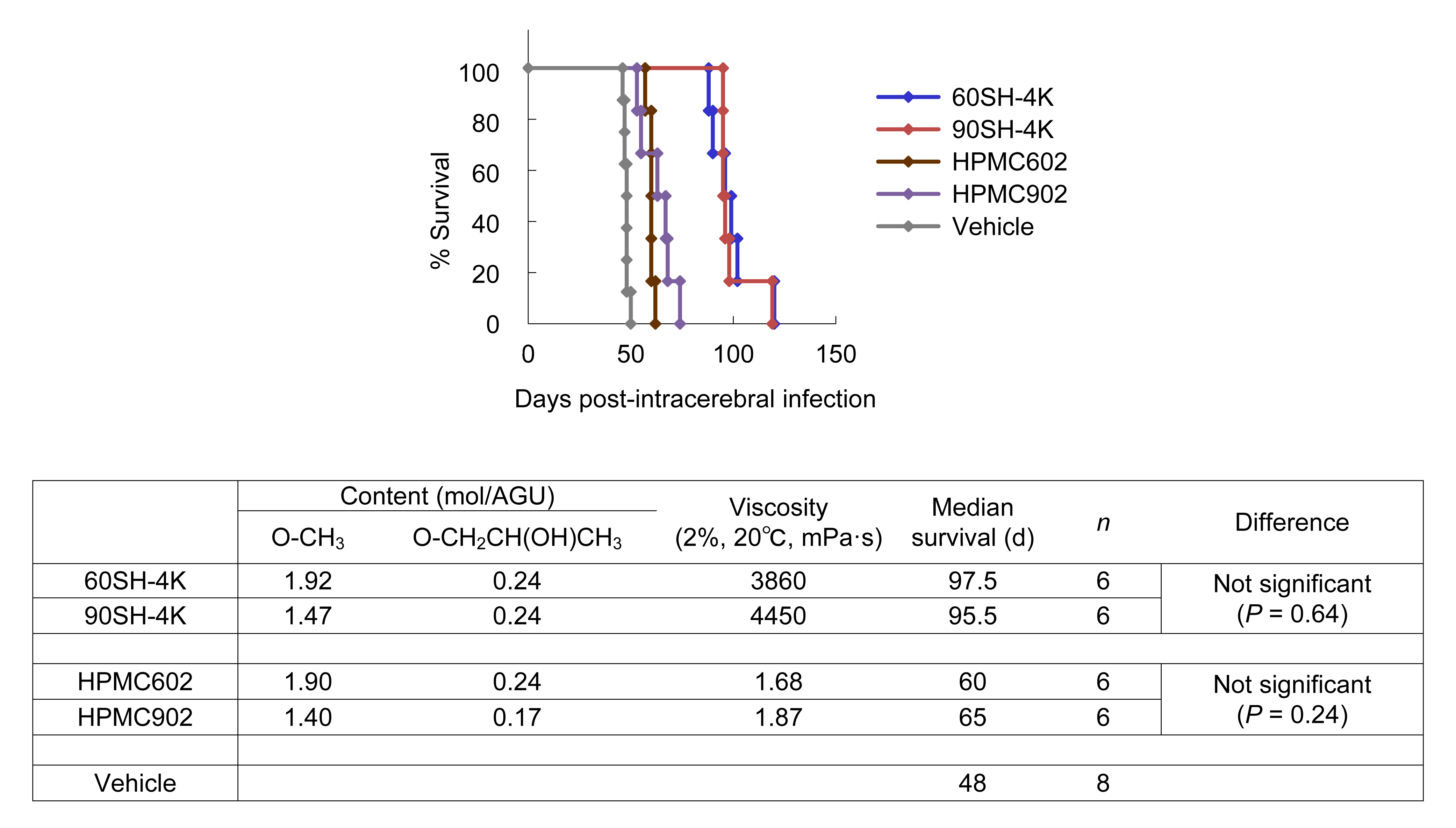

Supplement: S3 Fig — Survival analysis was performed in Tg7 mice intracerebrally infected with the 263K prion and treated 3 days pre-infection with a single subcutaneous injection (2 g/kg body weight) of HPMCs with similar viscosities but different modifications (60SH-4K vs. 90SH-4K and HPMC602 vs. HPMC902). (TIF) [file ppat.1006045.s003.tif]

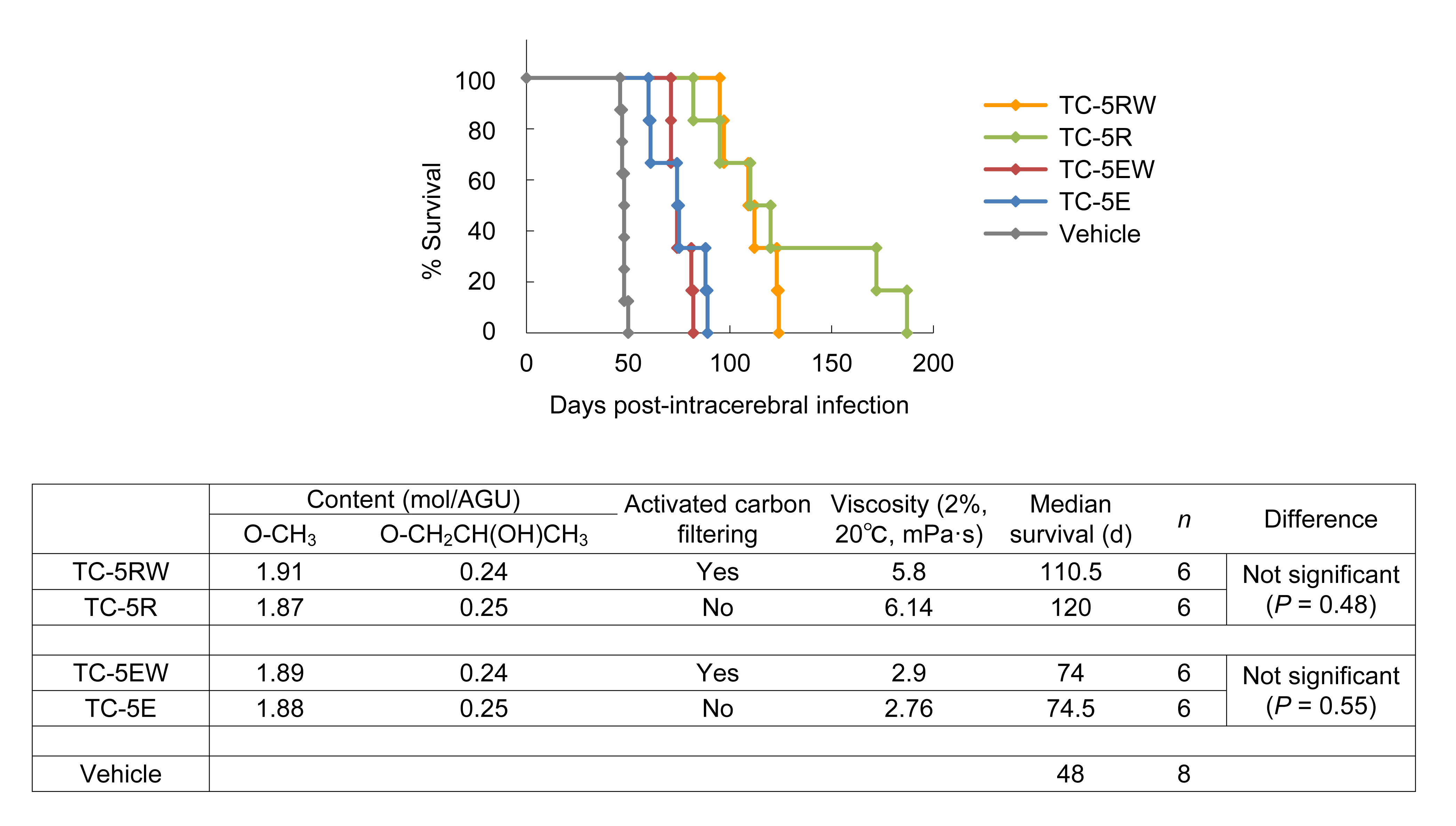

Supplement: S4 Fig — Survival analysis was performed as described in S3 Fig using either activated carbon-treated HPMCs or untreated HPMCs (TC-5RW vs. TC-5R and TC-5EW vs. TC-5E). (TIF) [file ppat.1006045.s004.tif]

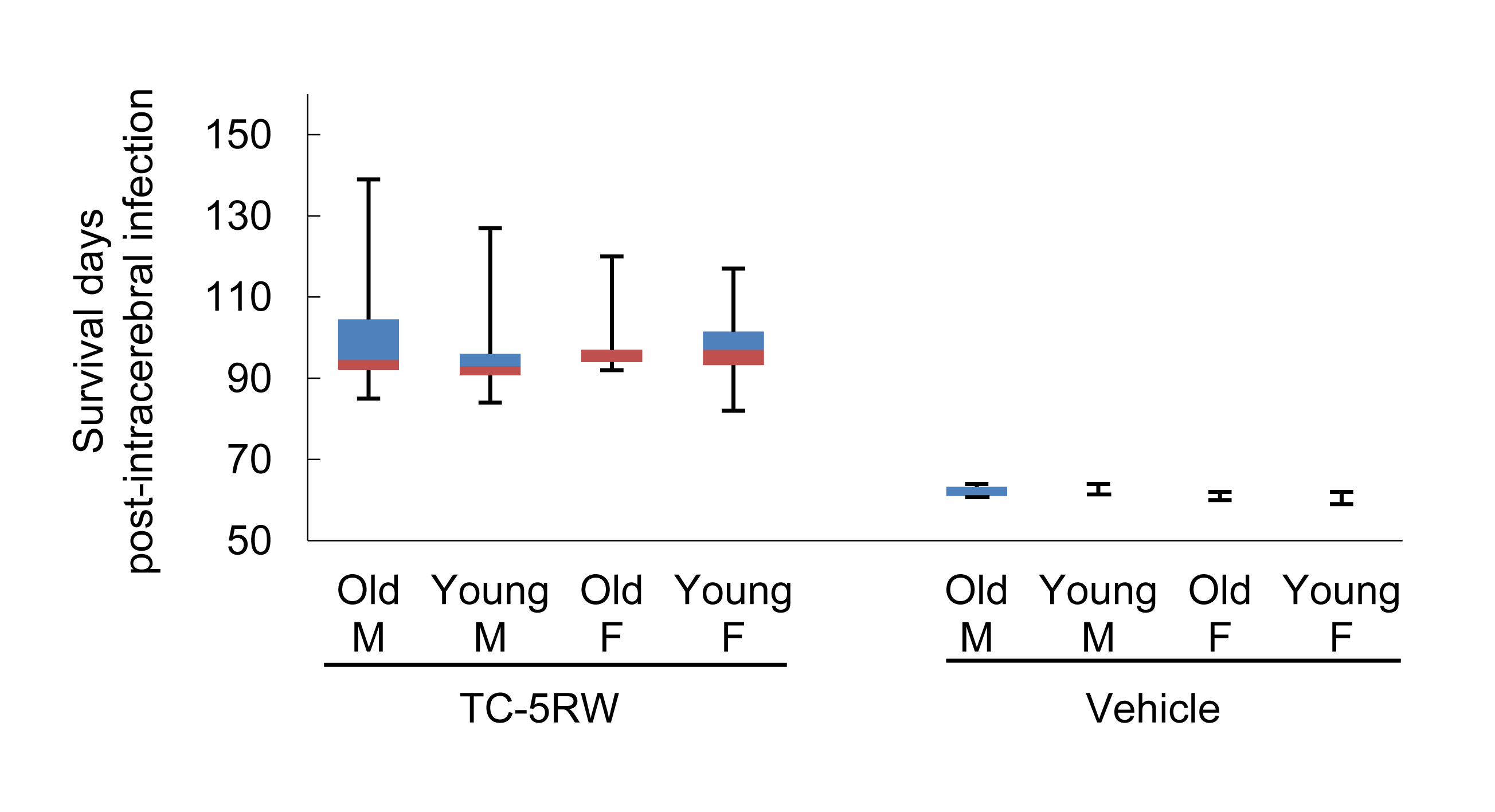

Supplement: S5 Fig — Box plots of survival periods in old (43–53 months) and young (6–10 months) male (M) and female (F) mice are shown. Tg7 mice were treated with a single intraperitoneal injection (1 g/kg body weight) 1 day before intracerebral infection with the 263K prion. No significant difference was observed among TC-5RW-treated groups (n = 5 or 6 for each group) or vehicle-treated groups (n = 6 for each group); log-rank test. (TIF) [file ppat.1006045.s005.tif]

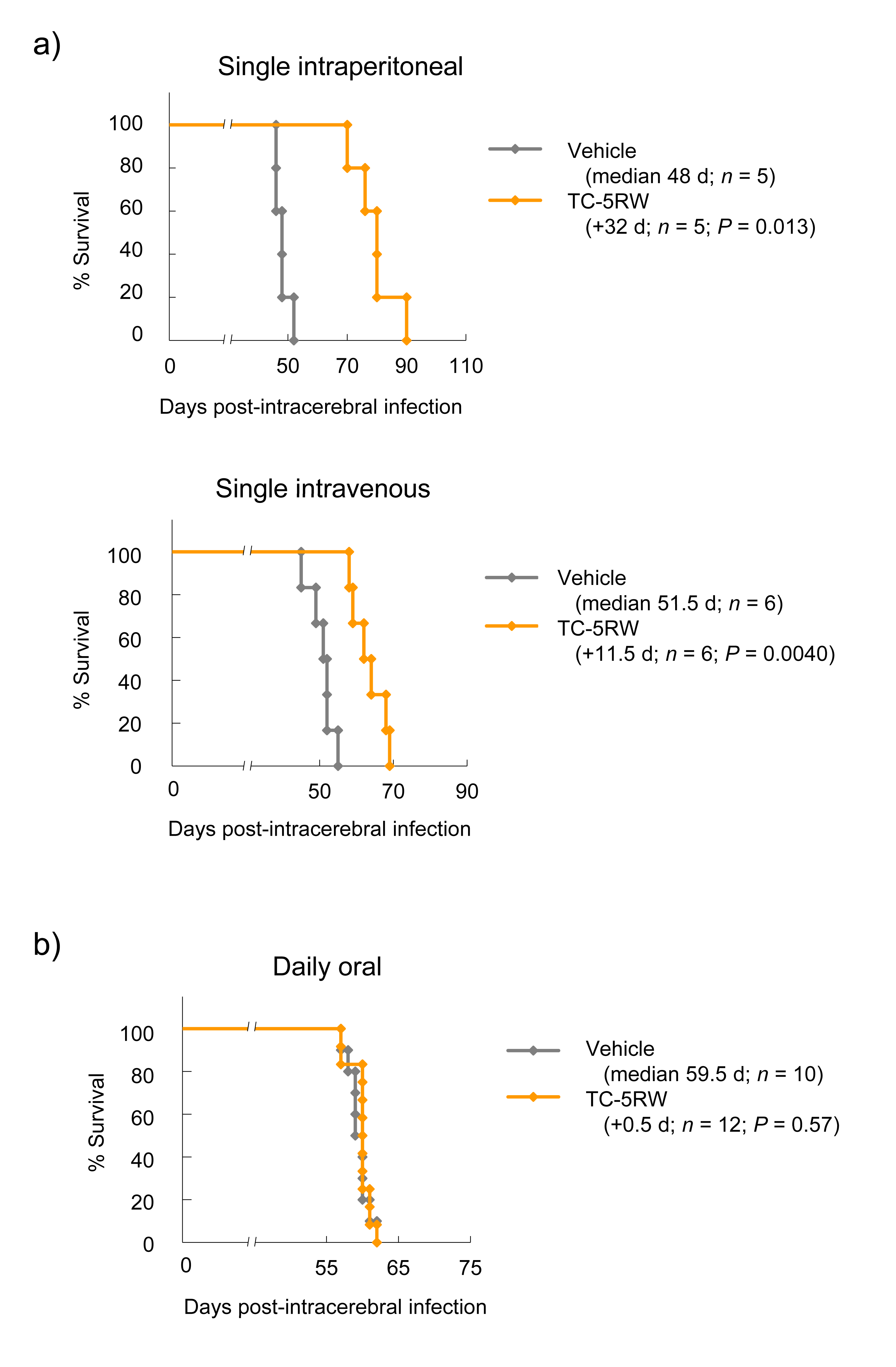

Supplement: S6 Fig — (a) Survival analysis was performed in Tg7 mice intracerebrally infected with the 263K prion and treated with TC-5RW via a single intraperitoneal infusion (1 g/kg body weight) immediately after infection or via a single intravenous injection (0.3 g/kg body weight) 6 h pre-infection. (b) Survival analysis was performed in Tg7 mice intracerebrally infected with the 263K prion and treated with a 15% TC-5RW-containing diet from 9 days pre-infection to akinetic terminal disease. (TIF) [file ppat.1006045.s006.tif]

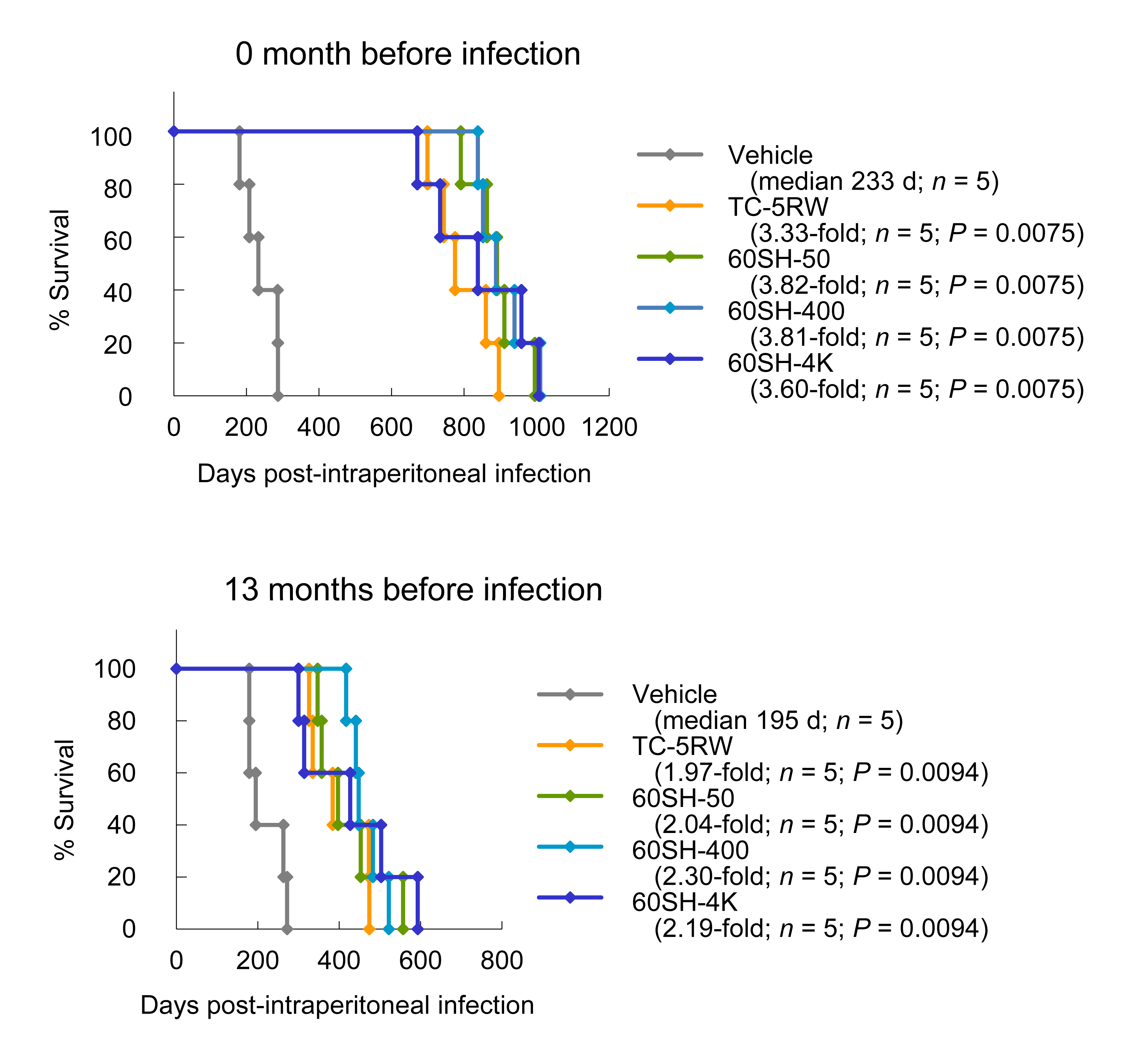

Supplement: S7 Fig — Survival analysis was performed in Syrian hamsters intraperitoneally infected with the 263K prion and treated with HPMCs via a single subcutaneous injection (4 g/kg body weight) at 0 or 13 months before infection. (TIF) [file ppat.1006045.s007.tif]

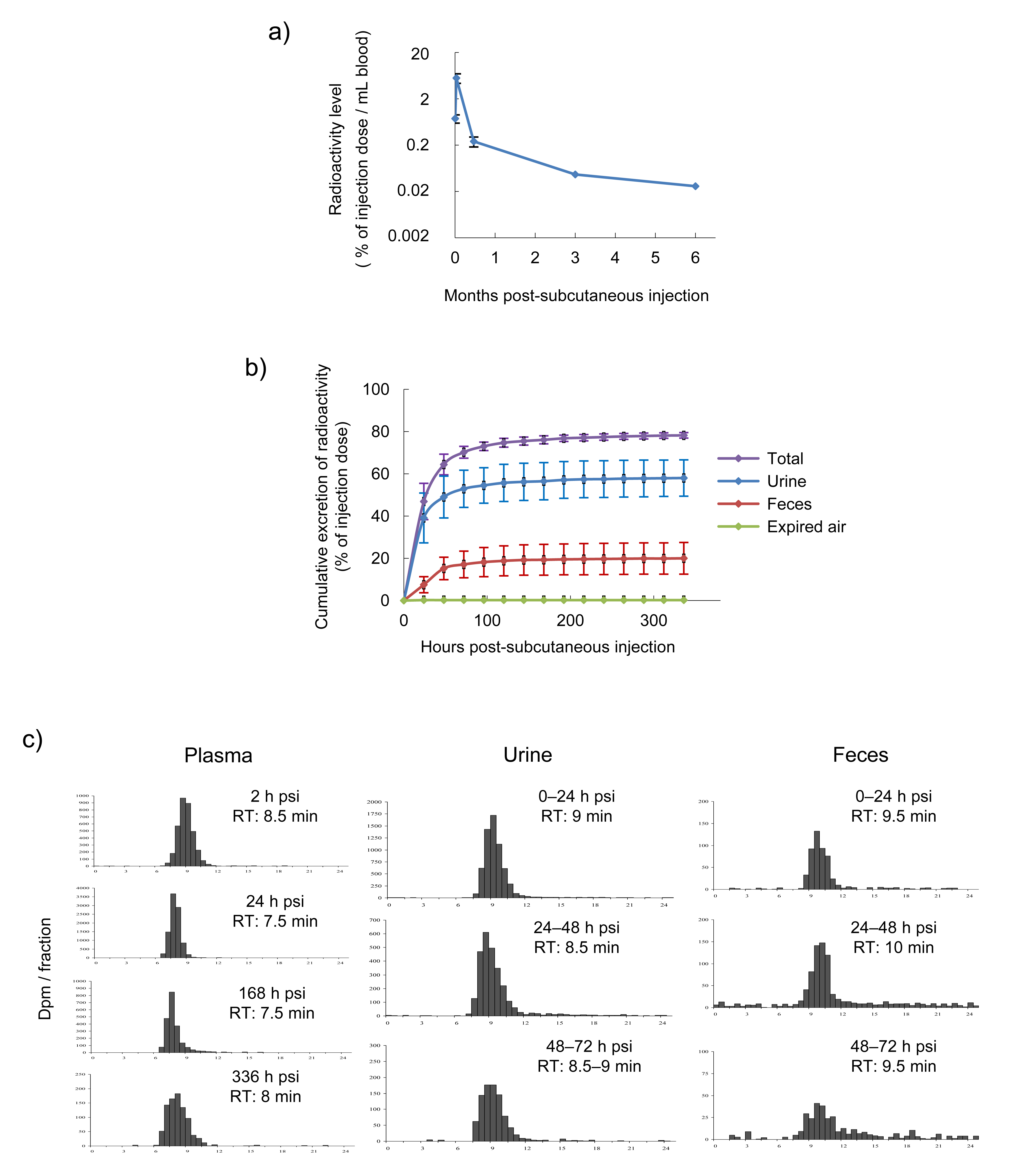

Supplement: S8 Fig — (a) Blood 14C-TC-5RW radioactivity levels in Tg7 mice that received a single subcutaneous injection of 14C-TC-5RW at 2 g/kg body weight. The mean and standard deviation are shown from technical triplicates at 3 or 6 months (n = 1) and from biological triplicates at other time points (n = 3). (b) Kinetics of 14C-TC-5RW radioactivity excretion by Tg7 mice that received a single subcutaneous injection of 14C-TC-5RW at 0.1 g/kg body weight. Total excretion radioactivity levels were determined from whole-body residual radioactivity levels. The mean and standard deviation are shown (n = 3). (c) 14C-TC-5RW molecular size profiles in the plasma, urine, and feces of Tg7 mice described in (b). GPC profiles of radioactivity were analyzed in plasma and excreta within 2 weeks and 3 days, respectively, of post-subcutaneous injection of 14C-TC-5RW (n = 1). RT, peak retention time; psi, post-subcutaneous injection. Methods: A designated amount of 14C-TC-5RW in saline was subcutaneously injected into the backs of mice. Blood or excreta samples were collected from the mice at designated time points. Urine and feces were collected in metabolic cages and expired CO2 was captured in 20% ethanolamine solution. Feces were dissolved with a scintillation solubilizer. The whole body was solubilized in heated 0.5 N NaOH/5% toluene solution to assay whole-body residual radioactivity. Portions of samples were mixed with a scintillation cocktail and radioactivity was assayed by liquid scintillation counting. GPC analysis was performed in a similar manner as described in the Materials and Methods section. (TIF) [file ppat.1006045.s008.tif]

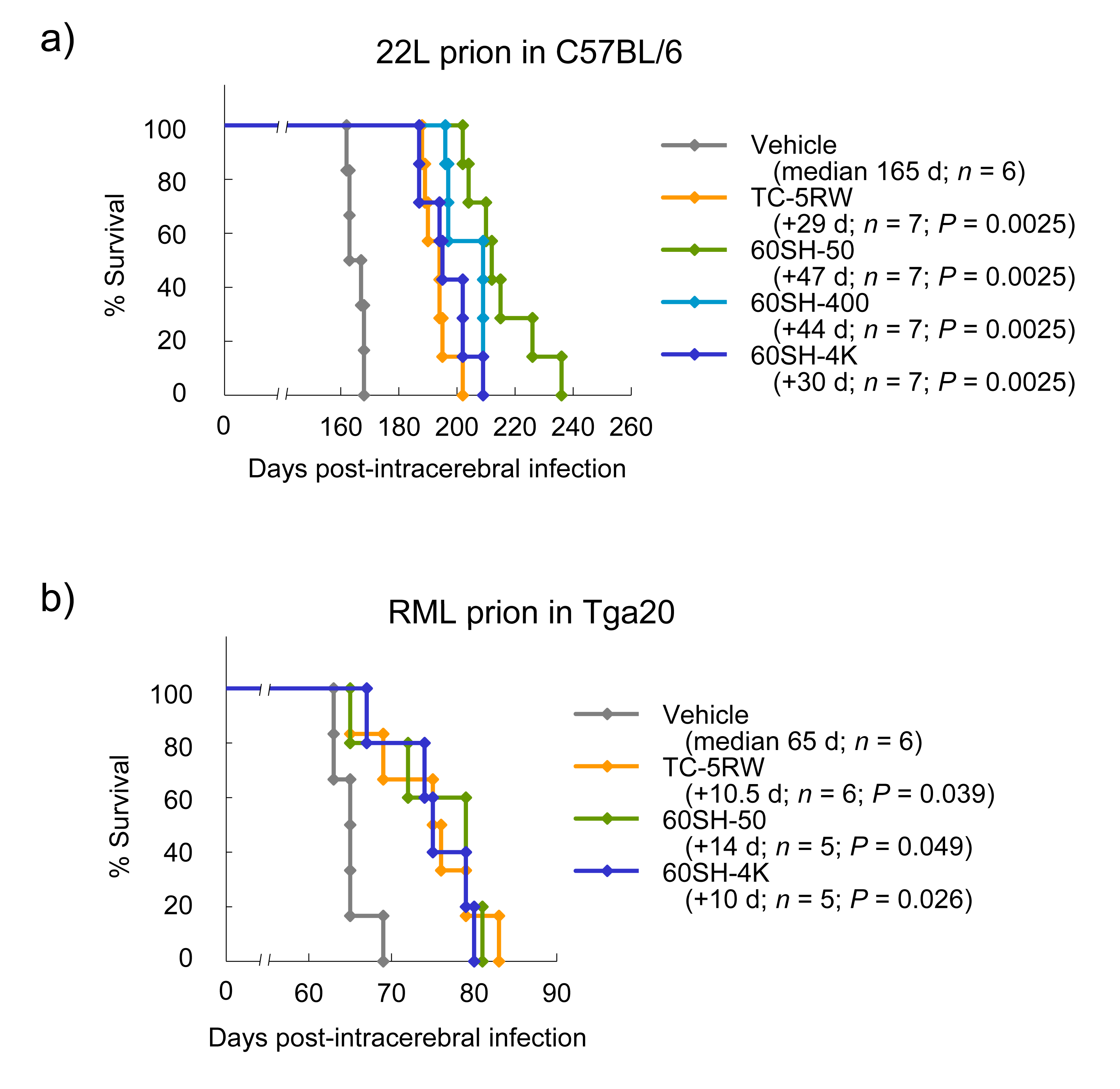

Supplement: S9 Fig — (a) Survival analysis of C57BL/6 mice intracerebrally infected with the 22L prion and treated with a single subcutaneous injection of CEs at 2 g/kg body weight 1 day before infection.(b) Survival analysis of Tga20 mice intracerebrally infected with the RML prion and treated with a single subcutaneous injection of CEs at 4 g/kg body weight immediately after infection. Tga20 mice overexpressing mouse PrPC [22] were kindly provided by Dr. Charles Weissmann of the Scripps Research Institute (La Jolla, CA, USA). (TIF) [file ppat.1006045.s009.tif]

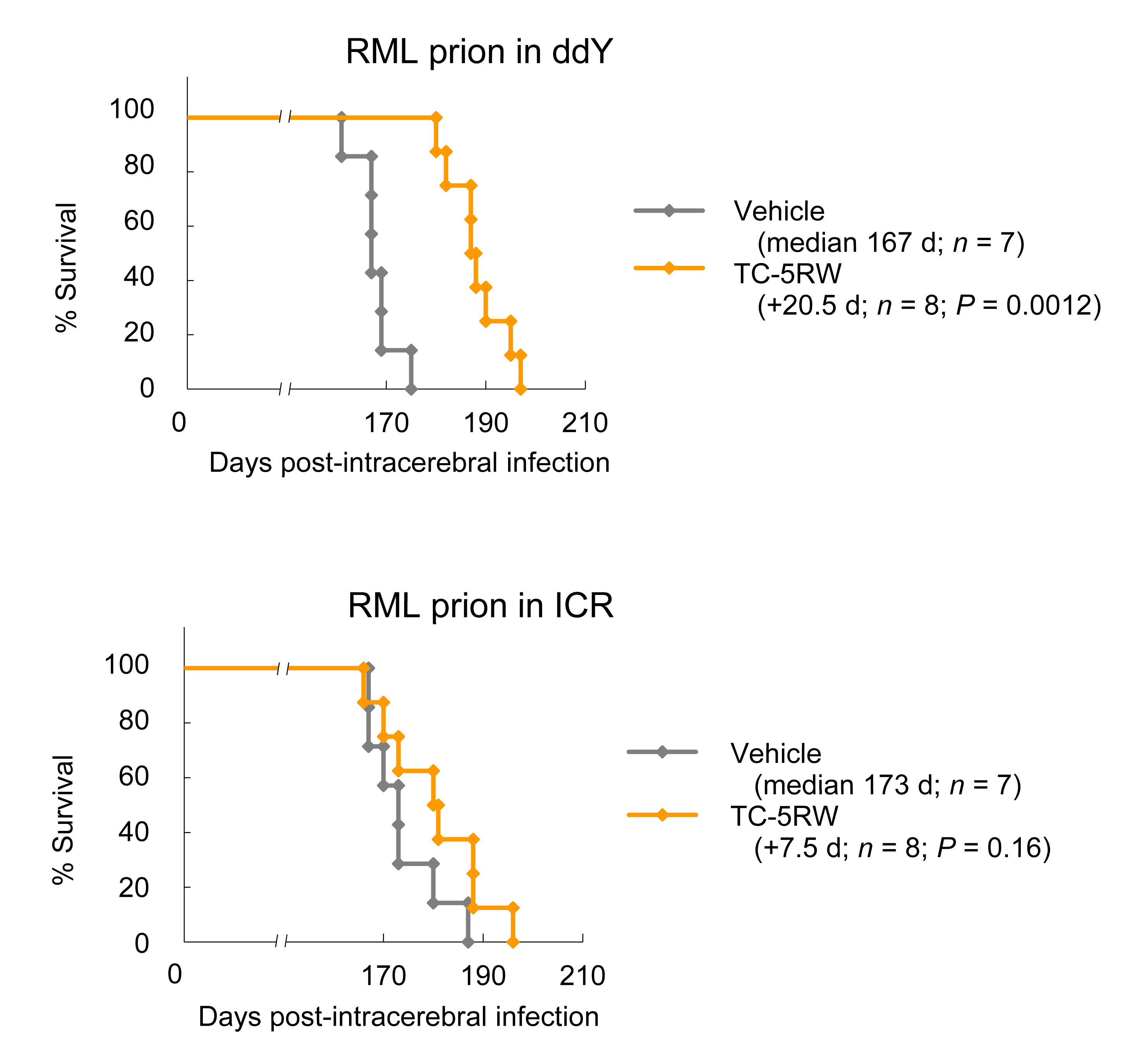

Supplement: S10 Fig — Survival analysis of ddY and ICR mice intracerebrally infected with the RML prion and treated with a single subcutaneous injection of TC-5RW (2.5 g/kg body weight) 1 day before infection. These mice were purchased from Japan SLC, Inc. (Hamamatsu, Japan). (TIF) [file ppat.1006045.s010.tif]

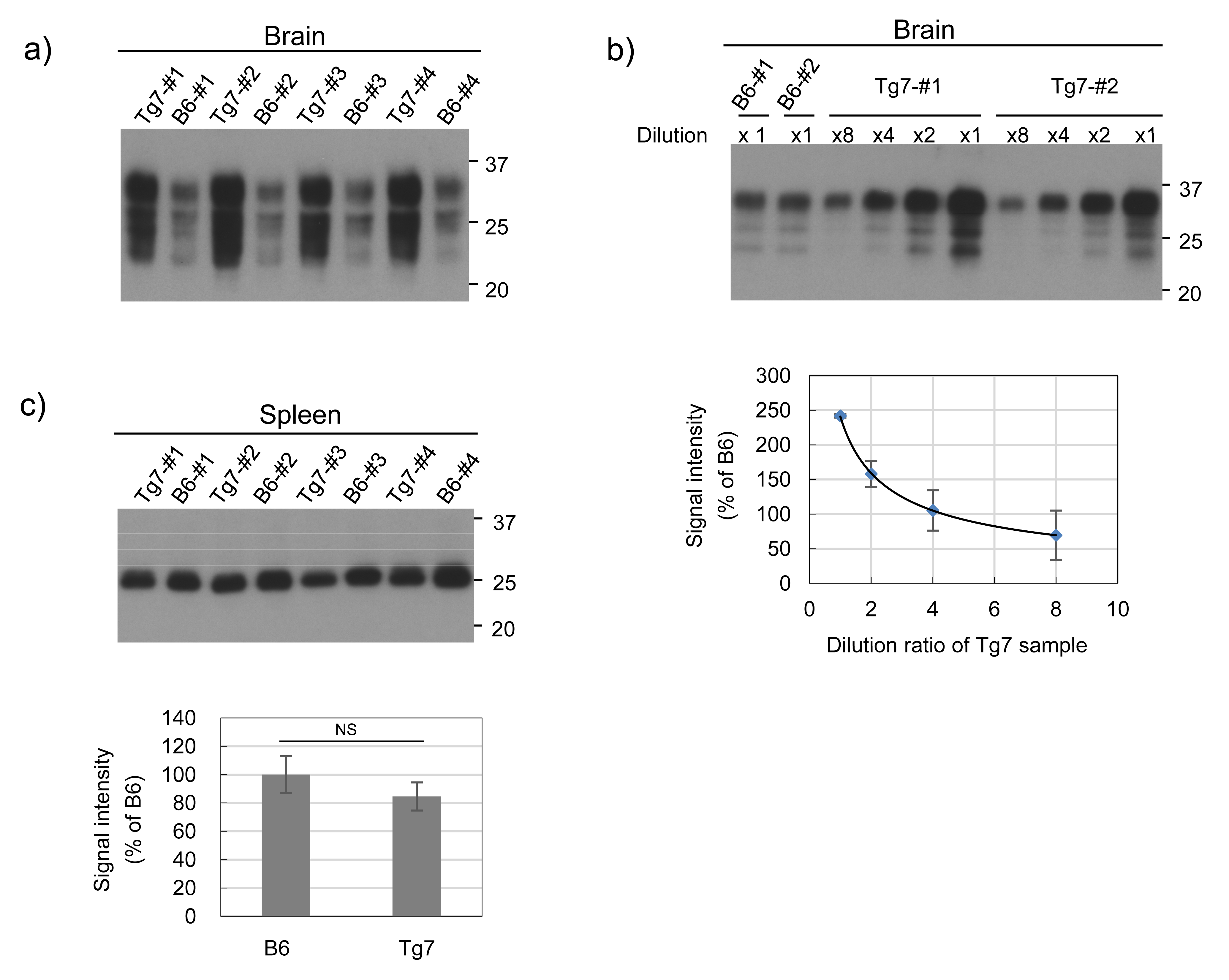

Supplement: S11 Fig — (a) PrPC levels in the brain from Tg7 and C57BL/6 (B6) mice. Brain homogenates containing 5 μg protein were analyzed by immunoblotting with SAF83 antibody (n = 4 for each mouse strain). Molecular size markers on the right indicate sizes in kDa. (b) Quantitative comparison of PrPC levels in the brain between Tg7 and B6 mice. Two of the Tg7 brain samples in (a) were diluted and the PrPC levels were compared with those of undiluted B6 brain samples by immunoblotting. A plot of relative signal intensities for Tg7 samples (% of B6) is shown against dilution ratios; the mean and standard deviation were obtained from technical triplicate analysis (n = 2 for each mouse strain). (c) PrPC levels in the spleen from Tg7 and B6 mice. Spleen homogenates containing 10 μg protein were analyzed by immunoblotting as described in (a) (n = 4 for each mouse strain). The mean and standard deviation of signal intensities are shown. NS not significant; t-test. (TIF) [file ppat.1006045.s011.tif]
