# Supplementary material for: A Single Subcutaneous Injection of Cellulose Ethers Administered Long before Infection Confers Sustained Protection against Prion Diseases in Rodents
Source: PLoS Pathog. 2016 Dec 14;12(12):e1006045. doi: 10.1371/journal.ppat.1006045 (PMC5156379; doi:10.1371/journal.ppat.1006045)
Supplement: S1 Table — Survival analyses were performed in 263K prion-infected Tg7 mice treated with a single dose of each sample at a designated timing, as described in the Materials and Methods section. Dextran and dextrin were provided by Meito Sangyo (Nagoya, Japan) and pullulan by Hayashibara (Okayama, Japan). Hydroxypropyl methyl ethers of these compounds were synthesized by Meito Sangyo. Other compounds were purchased from Sigma-Aldrich, Tokyo Chemical Industry, and Wako Pure Chemicals (Tokyo, Japan). (DOCX) [file ppat.1006045.s012.docx]

**S1　Table. Other tested polysaccharide ethers**

| Backbone structure | Compound name | Dose, injection site, and timing | Modifications | Size | Median survival  (% of vehicle control) | *n* | Difference  from vehicle control |
| --- | --- | --- | --- | --- | --- | --- | --- |
| Dextran | HP-M-dextran-2 | 2.5 g/kg sc  at day1 post-ici | M content: 2 mol/AGU  HP content: 0.27 mol/AGU | Average MW:  approximately 2000 Da | 100 | 7 | Not significant (*P* = 0.78) |
|  | HP-M-dextran-10 |  | M content: 2 mol/AGU  HP content: 0.24 mol/AGU | Average MW:  approximately 10,000 Da | 100 | 7 | Not significant (*P* = 1.00) |
|  | HP-M-dextran-70 |  | M content: 2.2 mol/AGU  HP content: 0.27 mol/AGU | Average MW:  approximately 70,000 Da | 101.7 | 7 | Not significant (*P* = 0.56) |
| Dextrin | HP-M-dextrin-2 |  | M content: 2.08 mol/AGU  HP content: 0.23 mol/AGU | Average MW:  approximately 2000 Da | 100 | 7 | Not significant (*P* = 0.62) |
|  | HP-M-dextrin-10 |  | M content: 2.16 mol/AGU  HP content: 0.2 mol/AGU | Average MW:  approximately 10,000 Da | 105 | 7 | Not significant (*P* = 0.18) |
|  | HP-M-dextrin-70 |  | M content: 2.2 mol/AGU  HP content: 0.22 mol/AGU | Average MW: approximately 70,000 Da | 100 | 7 | Not significant (*P* = 0.59) |
| Pullulan | HP-M-pullulan-2 |  | M content: 2.17 mol/AGU  HP content: 0.27 mol/AGU | Average MW: approximately 2,000 Da | 100 | 7 | Not significant (*P* = 0.97) |
|  | HP-M-pullulan-10 |  | M content: 2.21 mol/AGU  HP content: 0.22 mol/AGU | Average MW: approximately 10,000 Da | 101.7 | 7 | Not significant (*P* = 0.46) |
|  | HP-M-pullulan-70 |  | M content: 2.04 mol/AGU  HP content: 0.29 mol/AGU | Average MW: approximately 70,000 Da | 100 | 7 | Not significant (*P* = 0.80) |
| Chitin | HP-chitin | 0.6 g/kg ip  at day5 post-ici | HP content: 20% minimum | Not specified | 100 | 5 | Not significant (*P* = 0.94) |
| Chitosan | HP-chitosan-100 | 0.6 g/kg ip  at day5 post-ici | HP content: not specified | Average viscosity: 100 cps (0.5% solution at 20°C) | 102.2 | 5 | Not significant (*P* = 0.48) |
| Starch | Hydroxyethyl starch | 5 g/kg ip  at day5 post-ici | Hydroxyethyl content: 0.55 mol/AGU | Average MW: approximately 70,000 Da | 100 | 5 | Not significant (*P* = 0.56) |
| Cyclodextrin | M-beta-CD | 0.6 g/kg sc  at day5 post-ici | M content: 1.6–2.0 mol/AGU |  | 100 | 5 | Not significant (*P* = 0.99) |
|  | HP-beta-CD |  | HP content: 0.8 mol/AGU |  | 93.5 | 5 | Not significant (*P* = 0.51) |
|  | Acetyl-beta-CD |  | Acetyl content: 1.14 mol/AGU |  | 100 | 5 | Not significant (*P* = 0.99) |
|  | HP-gamma-CD | 2 g/kg sc  at day 5 post-ici | HP content: not specified |  | 93.5 | 5 | Not significant (*P* = 0.51) |
